# BMSC‐NFMC Model for Vascular Regulation and Interface Integration in Osteochondral Regeneration

**DOI:** 10.1002/advs.202505222

**Published:** 2025-06-23

**Authors:** Qian Zhou, Mengjie Hou, Baoshuai Bai, Yiwu Zhang, Yiwei Shen, Zenghui Jia, Yongqiang Guo, Guangdong Zhou, Xiaoqin Liang

**Affiliations:** ^1^ Plastic Surgery Institute Shandong Second Medical University Weifang Shandong 261053 P. R. China; ^2^ Department of Plastic and Reconstructive Surgery Shanghai Key Laboratory of Tissue Engineering Shanghai Ninth People's Hospital Shanghai Jiao Tong University School of Medicine Shanghai 200011 China; ^3^ Department of Orthopaedics Qilu Hospital of Shangdong University Centre for Orthopaedics Shandong University Jinan Shandong 250100 P. R. China; ^4^ Department of Orthopedics The 80th Group Army Hospital of PLA Weifang Shandong 261000 China

**Keywords:** BMSCs, interface integration, nanofibrous materials, osteochondral tissue engineering, vascularization regulation

## Abstract

The core challenge in osteochondral tissue engineering is achieving the dual objectives of precise vascularization regulation and effective interface integration. Current tissue‐engineering strategies have limitations in addressing these challenges. This study has regulated BMSC differentiation by optimizing the GT/PCL ratio and topological structure of nanofibrous materials, systematically comparing three different materials (r5G5P, a5G5P, and a7G3P), and employing a “rolling and folding” method in order to construct BMSC‐NFMC composite structures. This approach achieves effective vascular isolation between the bone and cartilage layers. After implantation in nude mice, the a5G5P group exhibits distinct natural osteochondral tissue structural characteristics, which become more stable after 8 weeks of in vivo culture. Transcriptome sequencing analysis reveals that under ischemic conditions, the a5G5P group effectively regulates cartilage formation by inhibiting the Rap1 pathway and subsequently activating the ERK pathway. In rabbit articular osteochondral defect repair experiments, the a5G5P group successfully regenerates complete articular osteochondral structures similar to those of the adjacent natural tissues. The BMSC‐NFMC structure can be used for both local and long‐segment osteochondral defect repair, providing broader possibilities for clinical applications.

## Graphical abstract

A novel strategy for integrated osteochondral tissue regeneration using BMSC‐NFMCs, this study has demonstrated the precise regulation of vascularization and effective interface integration using optimized nanofibrous materials and innovative construction methods.

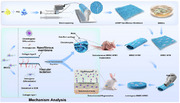

## 1 Introduction

The osteochondral interface represents a complex and precise tissue transition zone in the human body and plays an irreplaceable role in joint biomechanical functions.^[^ ^1^, ^2^, ^3^ ^]^ This interface consists of superficial hyaline cartilage, deep calcified cartilage, a subchondral bone plate, and cancellous bone, forming a highly integrated biological composite in both structure and function.^[^ ^4^, ^5^, ^6^, ^7^, ^8^ ^]^ The hyaline cartilage region is almost avascular, primarily obtaining nutrients through diffusion, whereas the bone tissue is highly vascularized, relying on vascular networks for nutrient supply and waste removal. This significant histological difference makes the osteochondral interface a critical region for biomechanical load transfer and also a natural vascular gradient structure that maintains the unique microenvironment of each tissue.^[^ ^9^, ^10^, ^11^, ^12^ ^]^ However, because of its special structure and limited endogenous repair capacity, once damaged, it is difficult to restore naturally, which presents major challenges for clinical treatment. Globally, over 50 million people are affected by osteochondral defect‐related diseases, which not only severely diminish the quality of life of patients but also impose an enormous economic burden on healthcare systems.^[^ ^13^, ^14^ ^]^

Tissue engineering, as a cutting‐edge therapeutic strategy combining biomaterials, cells, and growth factors, holds promise for achieving functional osteochondral composite tissue regeneration.^[^ ^15^, ^16^, ^17^, ^18^, ^19^, ^20^ ^]^ However, osteochondral tissue engineering faces two fundamental challenges: precise regulation of vascularization and effective interface integration. The ideal cartilage region should maintain a relatively ischemic microenvironment, while the bone region requires sufficient vascularization to support its growth and function.^[^ ^9^, ^12^, ^21^, ^22^ ^]^ Simultaneously, a stable and gradual transition zone must be formed between these two tissues to ensure biomechanical properties and structural integrity.^[^ ^10^, ^11^, ^23^ ^]^ These two objectives often counteract each other, as factors promoting bone tissue vascularization typically interfere with cartilage formation and maintenance, whereas signals supporting cartilage formation may inhibit bone development and vascularization.

Existing tissue‐engineering strategies have limitations in resolving these contradictions. Single‐layer material structures, though simple to prepare, cannot meet the differentiated needs of both tissues; bilayer gradient structures mimic interface transitions through physical or chemical methods but suffer from insufficient interface bonding strength and a tendency for delamination; multilayer composite structures achieve functional zoning but have significant limitations in vascularization control.^[^ ^24^, ^25^ ^]^ Currently, common material combination methods such as direct stacking, chemical crosslinking, and 3D printing often result in inadequate interface bonding, which affects the overall repair efficacy.^[^ ^15^, ^26^, ^27^, ^28^ ^]^ These methods typically struggle to precisely control vascular invasion from the bone region to the cartilage region, leading to cartilage tissue degeneration or heterotopic ossification.

Cell selection is a key consideration in osteochondral tissue engineering. Bone marrow mesenchymal stem cells (BMSCs) have emerged as ideal seed cells owing to their multidirectional differentiation potential, self‐renewal capacity, and abundant availability.^[^ ^29^ ^]^ Research has shown that the direction of BMSC differentiation is regulated by multiple factors, including the physicochemical properties of the material (such as stiffness and surface morphology), biochemical signals (such as growth factors), and physical signals (such as mechanical forces and oxygen concentrations).^[^ ^30^, ^31^, ^32^ ^]^ Notably, a low‐oxygen environment (1–5% O₂) favors chondrogenesis, whereas normal oxygen tension (≈20% O₂) promotes osteogenic differentiation.^[^ ^33^, ^34^ ^]^ This oxygen gradient dependency provides important insights into osteochondral interface design.

Regarding material selection, electrospun nanofibrous materials have demonstrated enormous potential in tissue engineering due to their three‐dimensional network structure similar to natural extracellular matrix, large surface area, and high porosity.^[^ ^35^ ^]^ Specifically, by regulating the fiber diameter, orientation, surface morphology, and composition ratio, cell behavior and tissue formation can be precisely adjusted.^[^ ^36^, ^37^, ^38^ ^]^ Composite materials of gelatin (GT) and polycaprolactone (PCL) combine the advantages of gelatin, which provides good biocompatibility and bioactive sites, whereas PCL offers appropriate mechanical strength and controlled degradability.^[^ ^39^ ^]^ Additionally, the fiber orientation structure can guide cell arrangement through contact guidance effects, thereby influencing tissue formation patterns.^[^ ^40^, ^41^ ^]^

**Figure 1:**
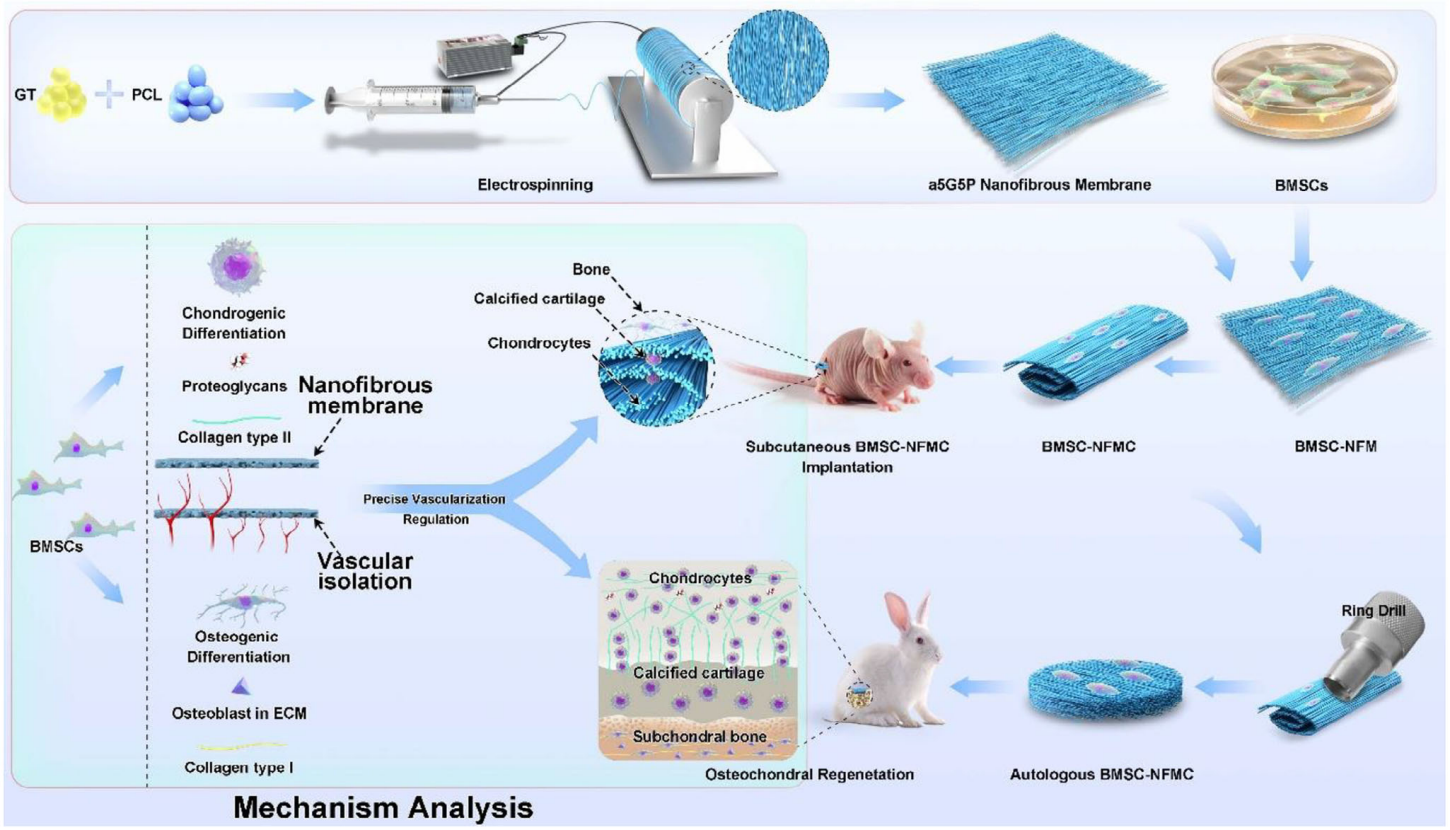
Schematic illustration of the BMSC‐NFMC model for integrated osteochondral tissue regeneration.

Based on a deep understanding of osteochondral interface characteristics and also the limitations of existing repair strategies, this study aimed to develop an innovative tissue engineering approach. First, nanofibrous membranes with different GT/PCL ratios and orientations were prepared using electrospinning, and the differences in regard to the physicochemical and biological properties of the randomly oriented 5:5 GT/PCL (r5G5P), aligned 5:5 GT/PCL (a5G5P), and aligned 7:3 GT/PCL (a7G3P) materials were determined. BMSCs were seeded onto these nanofibrous membranes to construct BMSC‐nanofibrous membranes (BMSC‐NFMs). The adhesion, proliferation, and multidirectional differentiation capabilities of BMSCs cultured on these materials were analyzed. Next, the BMSC‐NFMs were rolled and folded into four layers to form BMSC‐nanofibrous membrane composites (BMSC‐NFMCs) and subcutaneously transplanted into nude mice to explore the feasibility of osteochondral integrated regeneration. Finally, the two groups with the most significant effects in the subcutaneous experiments in nude mice (a7G3P‐BMSC‐NFMC and a5G5P‐BMSC‐NFMC) were selected for in situ repair of rabbit articular osteochondral defects to evaluate the effectiveness of the BMSC‐NFMC model in osteochondral defect repair. By precisely regulating the material characteristics and construction methods, this approach simultaneously achieves the dual objectives of precise vascularization control and effective interface integration, providing a new solution for the clinical treatment of osteochondral defects (**Figure** **1**).

## 2 Results

### 2.1 Characterization of Nanofibrous Membranes with Different Structures and Compositions

**Figure 2:**
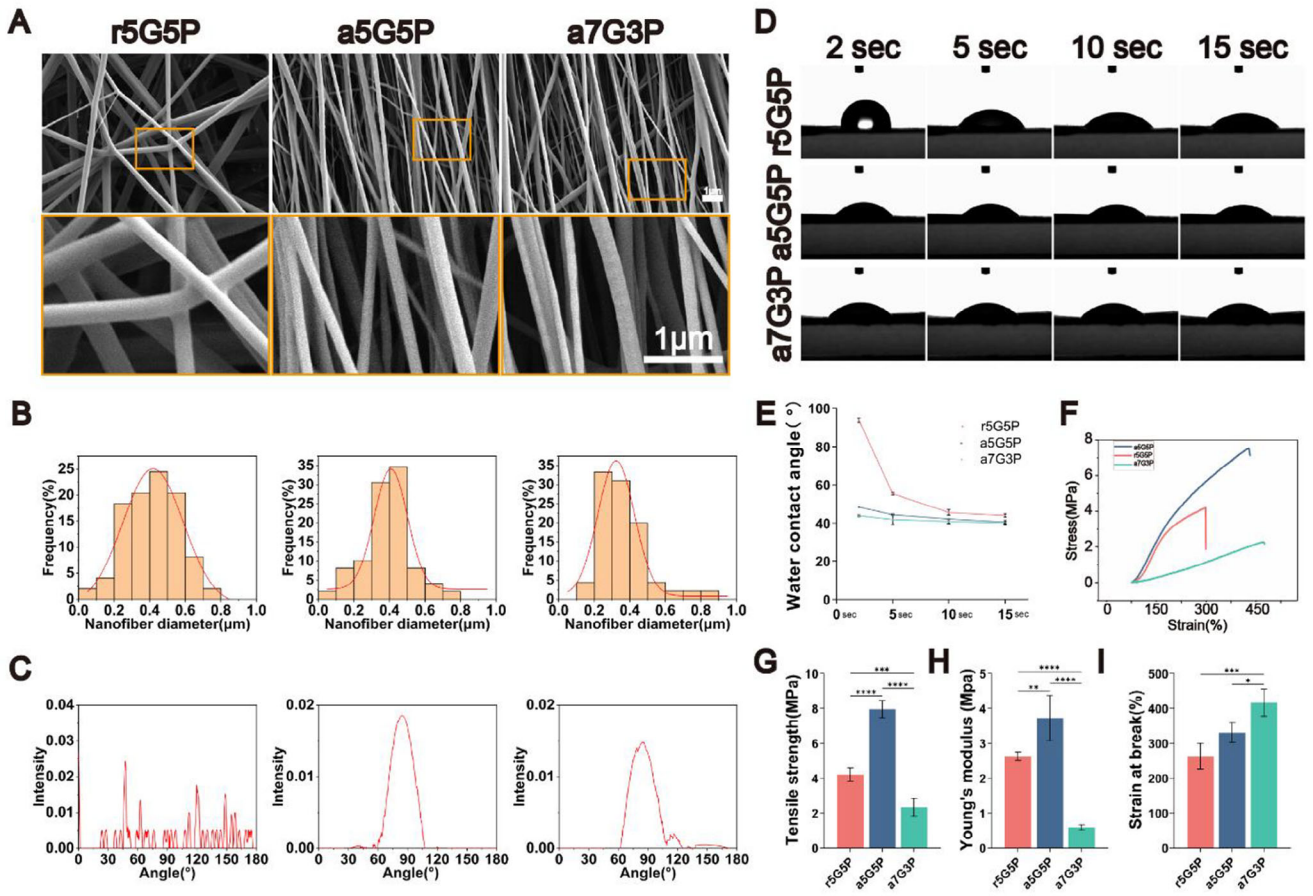
Characterization of nanofibrous membranes with different structures and compositions. A) Scanning electron microscopy images, B) nanofiber diameter distribution, C) fiber angle distribution, and D,E) contact angle analysis of the three types of nanofibrous membranes. F) Stress–strain curves, G) Young's modulus, H) tensile strength and I) elongation at break of all nanofibrous membrane groups. Data are presented as mean ± SD (*n* = 4 for mechanical tests; *n* = 40–50 for fiber diameter and angle measurements; *n* = 3 for contact angle analysis). Statistical significance was determined using one‐way ANOVA with Tukey's post‐hoc test for G, H, and I. Abbreviations: GT, gelatin; PCL, polycaprolactone. ^*^ *p* < 0.05, ^**^ *p* < 0.01, ^***^ *p* < 0.001, ^****^ *p* < 0.0001.

Scanning electron microscopy (SEM) images revealed that all the three nanofibrous membranes exhibited typical fibrous structures. The diameter, size, and distribution of the nanofibers significantly affected cell adhesion and growth. Analysis of nanofiber diameter distribution showed that the average diameters of r5G5P, a5G5P, and a7G3P fibrous membranes were 415 ± 145, 385 ± 125, and 367 ± 157 nm, respectively. Past research has demonstrated that fiber diameters in the range of 0.2–0.6 µm are conducive to cell adhesion and spreading. The diameters of all of the three fibers displayed normal distribution characteristics, with peaks concentrated in the 0.2–0.6 µm range, and no statistically significant differences were observed (**Figure** **2**A,B). Furthermore, fiber angle distribution analysis indicated that r5G5P presented multiple irregular peaks, suggesting a random fiber orientation, whereas the a5G5P and a7G3P groups displayed single and sharp peaks, confirming that these two fiber types possessed a well‐aligned orientation (Figure 2C). These findings suggest that the preparation method affects the surface morphology of the nanofibrous membranes.

Material wettability is a key factor affecting cell adhesion and proliferation. The contact angle test results showed that all three nanofibrous membranes exhibited decreasing trends over time (2–15 s). Among them, r5G5P had the highest initial contact angle (≈90°), whereas a5G5P and a7G3P had lower initial contact angles (≈40–50°). Material surfaces with contact angles in the range of 40–90° are favorable for protein adsorption and cell adhesion. After 15 s, the contact angles of all three membranes stabilized at ≈40°. The results indicated that aligned fibrous membranes were more hydrophilic than randomly oriented fibrous membranes, which was related to changes in the surface energy caused by the regular arrangement of fibers (Figure 2D,E).

Next, the mechanical properties of the nanofibrous membranes were evaluated (Figure 2F–I). The stress–strain curves showed that a5G5P had the steepest curve slope, thus indicating its highest stiffness. The performed quantitative analysis of the mechanical properties revealed that a5G5P had a significantly higher Young's modulus (≈8 MPa) and tensile strength (≈4 MPa) than the other two groups (Figure 2F–I). Research has shown that Young's modulus of a material can influence the direction of stem cell differentiation. The high Young's modulus of a5G5P approaches the hardness range of natural bone tissue, and this mechanical characteristic is conducive to the osteogenic differentiation of BMSCs on the material surface. Regarding the elongation at break, a7G3P exhibited the highest value (≈400%), followed by a5G5P (≈300%), while r5G5P had the lowest value (≈250%). The higher extensibility and moderate Young's modulus of a7G3P were more favorable for vascularization and ossification processes, a characteristic that will be further verified in subsequent experiments. These results suggested that the aligned fiber orientation structure and PCL content significantly influenced the mechanical properties of the materials. A higher PCL content (50%) combined with an aligned orientation structure can significantly enhance the strength and stiffness of the material, whereas an increase in the gelatin content (70%) is beneficial for improving the material extensibility.

### 2.2 Biocompatibility Assessment of Nanofibrous Membranes with Different Structures and Compositions

**Figure 3:**
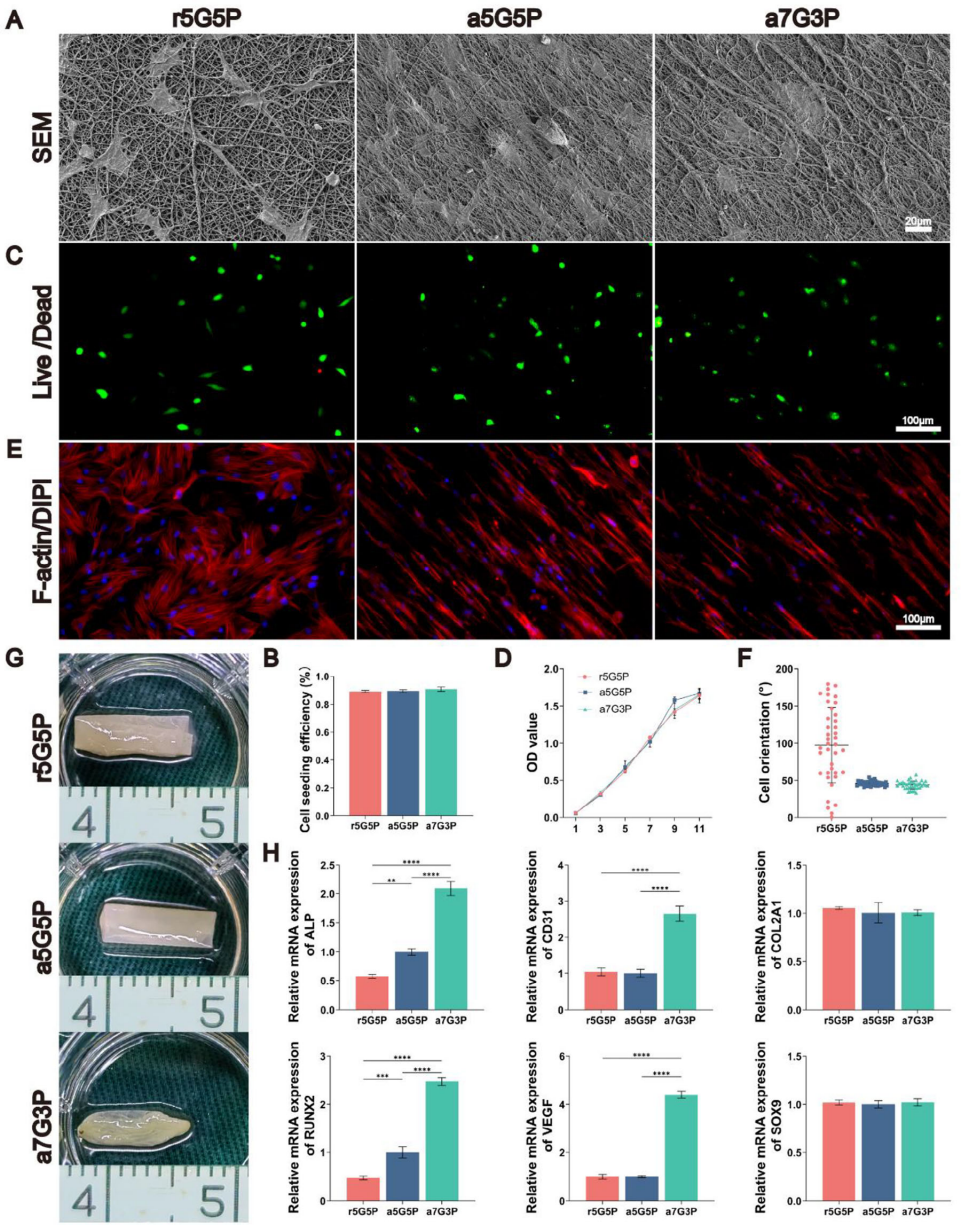
Biocompatibility assessment of nanofibrous membranes and construction of BMSC‐NFMC. A) Scanning electron microscopy images of BMSC adhesion on the nanofibrous membranes after 24 h of cell seeding, B) cell seeding efficiency, C,D) live/dead cell staining showing cell viability, and E,F) cytoskeletal protein (F‐actin) and nuclei (DAPI) staining showing the cell morphology and alignment after 3 days of culture. G) Construction process of BMSC‐NFMC using the “rolling and folding” method. H) RT‐qPCR analysis of osteogenesis‐related genes (*ALP* and *RUNX2*), angiogenesis‐related genes (*VEGF* and *CD31*) and chondrogenesis‐related genes (*SOX9* and *COL2A1*) using *GAPDH* as the reference gene. Data are presented as mean ± SD (*n* = 5 for cell seeding efficiency and cell viability tests; *n* = 3 for RT‐qPCR analysis). Statistical significance was determined using one‐way ANOVA with Tukey's post‐hoc test. Abbreviations: BMSCs: bone marrow stromal stem cells. ^*^ *p* < 0.05, ^**^ *p* < 0.01, ^***^ *p* < 0.001, ^****^ *p* < 0.0001.

The physicochemical properties of the nanofibers directly influenced the initial cell attachment efficiency. The biocompatibility assessment results indicated that BMSCs demonstrated good adhesion to the surface of the nanofibrous membranes in all of the groups after 24 h of cell seeding. SEM revealed tight interactions between the cells and fibrous membranes, with clearly visible pseudopodial structures (**Figure** **3**A). Cell counting analysis showed that the BMSC seeding efficiency reached ≈90% in all groups, with no significant differences between the groups (Figure 3B). This is closely related to the surface wettability and nanoscale surface morphology of the materials, which adsorb extracellular matrix proteins onto the fiber surface, thereby promoting cell adhesion. The live/dead cell staining results demonstrated that the BMSCs on all three nanofibrous membranes exhibited good viability 24 h after cell seeding, with very few dead cells (red) (Figure 3C). CCK8 proliferation analysis further confirmed that BMSCs in all groups showed similar proliferation trends, with cell numbers continuously increasing with extended culture time (1–11 days) (Figure 3D).

The aligned fiber orientation structure significantly influenced the cell morphology and arrangement through contact guidance. Double staining of cytoskeletal proteins (F‐actin, red) and nuclei (DAPI, blue) after 3 days of culture showed (Figure 3E) that on aligned nanofibrous membranes (a5G5P and a7G3P), BMSCs displayed a distinct spindle‐shaped morphology, with cells significantly elongated along the fiber direction and arranged in an orderly manner. Quantitative analysis confirmed this observation (Figure 3F). In contrast, on the randomly oriented r5G5P nanofibrous membranes, the cells exhibited a multidirectional extension morphology without obvious patterns in their arrangement. These results indicate that aligned nanofibrous structures can effectively induce the directional growth and arrangement of BMSCs.

### 2.3 In Vitro Construction of BMSC‐NFMC Model and Assessment of Osteogenic, Angiogenic, and Chondrogenic Potential

All of the groups of BMSC‐NFMCs cultured before transplantation exhibited a smooth, translucent appearance, with samples from the a7G3P group showing slight contractions (Figure 3G). Hematoxylin and eosin (H&E) staining revealed that the cells were evenly distributed on the membrane surface of the BMSC‐NFMCs from each group (Figure S2B, Supporting Information).

In order to systematically evaluate the influence of the NFMC model on the differentiation potential of BMSCs, we conducted RT‐qPCR analysis of multiple differentiation‐related genes in samples cultured in vitro for seven days (Figure 3H). The results indicate that regarding osteogenic‐related genes, the expression levels of *ALP* and *RUNX2* in the aligned nanofibrous membrane groups (a5G5P and a7G3P) were significantly higher than those in the randomly oriented nanofibrous membrane group (r5G5P) (*p* < 0.001). Notably, the expression levels seen in the a7G3P group were the most significant, with *ALP* expression approximately four times that of the r5G5P group and *RUNX2* expression approximately five times that of the r5G5P group (*p* < 0.0001). The biological signals provided by the high gelatin content (70%) synergistically interacted with the mechanical characteristics of the material to promote the joint expression of osteogenic‐ and angiogenic‐related genes.

In the analysis of angiogenesis‐related genes, the expression levels of *CD31* and *VEGF* in the a7G3P group were found to be significantly higher than those in the other two groups (*p* < 0.0001), ≈2.5 times and four times that in the r5G5P group, respectively. This suggests that the aligned fiber structure with high gelatin content not only promotes osteogenic differentiation but also enhances the angiogenic potential. Additionally, regarding chondrogenic‐related genes (*COL2A1* and *SOX9*), the expression levels were similar among the three sample groups, with no statistically significant differences observed (*p* > 0.05), indicating that the structure and composition of the materials had relatively minor effects on the chondrogenic differentiation potential of BMSCs in the in vitro environment.

In summary, the gene expression analysis results systematically elucidated the regulatory effects of the different scaffold structures and compositions on the multidirectional differentiation potential of BMSCs in an in vitro environment. Among them, the aligned fiber structure, particularly with a high proportion of gelatin content, not only significantly enhanced the osteogenic differentiation capacity of BMSCs, but also promoted the expression of angiogenic‐related genes.

### 2.4 Comprehensive Osteochondral Tissue Regeneration Using the BMSC‐NFMC Model

BMSC‐NFMCs from each group were subcutaneously transplanted into nude mice and cultured for 4–8 weeks to evaluate the feasibility of achieving comprehensive osteochondral regeneration in an ectopic microenvironment. Macroscopically, the a5G5P group gradually transformed from white tissue with partially calcified surfaces to completely calcified bone‐like tissue. The samples from the r5G5P group exhibited a smooth, milky‐white appearance after 4 weeks of culture. However, after eight weeks of subcutaneous culture, the surface changed to a rough, red appearance. In addition, the surfaces of the a7G3P samples gradually changed from smooth yellow to red and granular.

**Figure 4:**
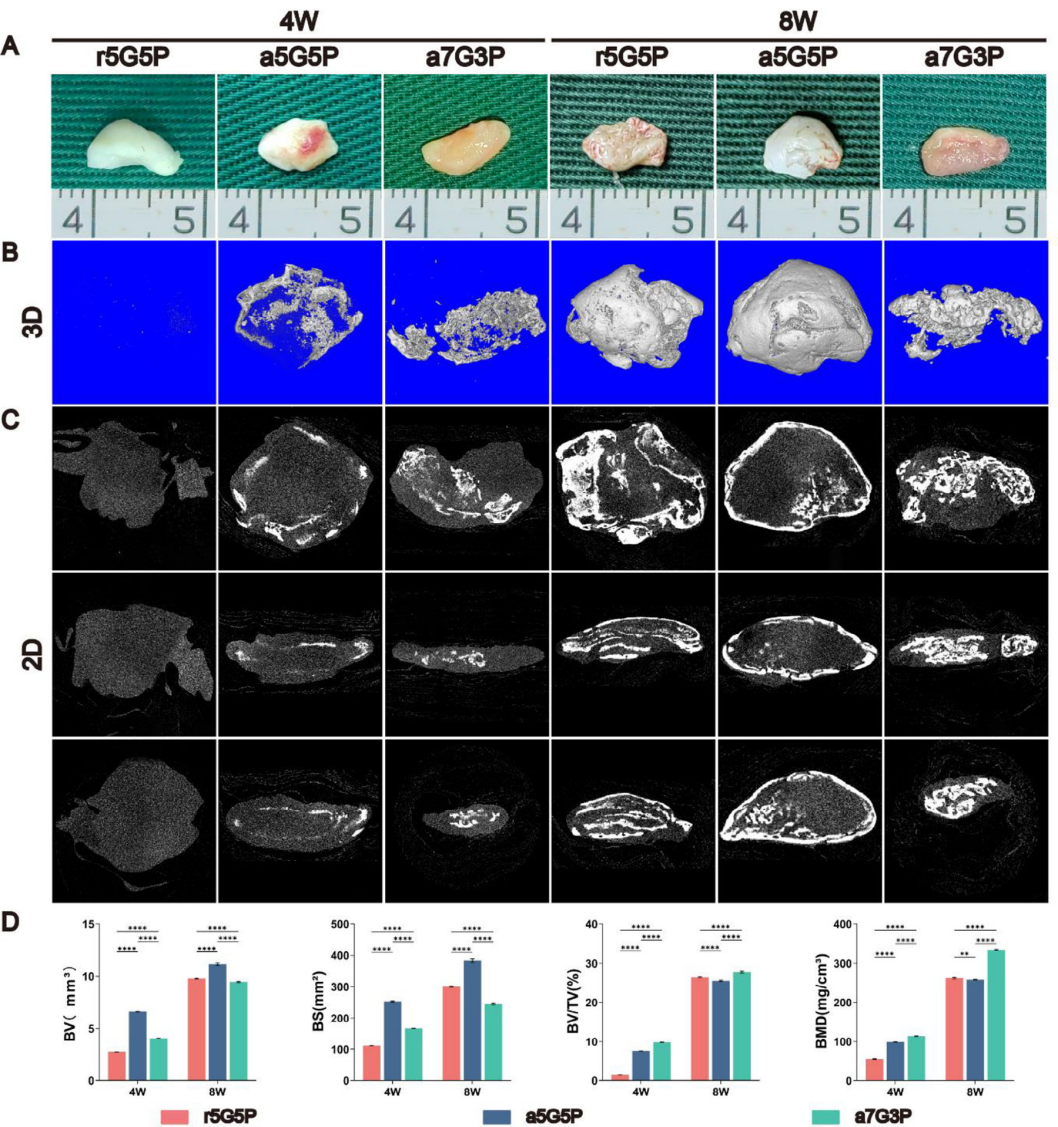
Macroscopic view and Micro‐CT assessment of the BMSC‐NFMC subcutaneously implanted in nude mice for 4 and 8 weeks. A) Macroscopic view and B,C) Micro‐CT images of constructs from groups a5G5P, r5G5P, and a7G3P. D) Quantitative analysis of bone volume (BV), bone surface (BS), bone volume fraction (BV/TV), and bone mineral density (BMD) from Micro‐CT analyses. Values are presented as mean ± standard deviation, *n* = 3. Statistical significance was determined using two‐way ANOVA with Bonferroni's post‐hoc test. ^*^ *p* < 0.05, ^**^ *p* < 0.01, ^***^ *p* < 0.001, ^****^ *p* < 0.0001.

The bone formation efficacy of BMSC‐NFMCs from each group was assessed using 2D and 3D Micro‐CT images, along with statistical analysis of bone volume (BV), bone surface (BS), bone volume fraction (BV/TV), and bone mineral density (BMD) (**Figure** **4**). Samples from the a5G5P group formed a uniformly arranged bone tissue in the outer layer, covering the largest area. In contrast, the regenerated bone tissue in the r5G5P and a7G3P groups showed a disordered and uneven distribution. The r5G5P group had the lowest bone volume at 4 weeks, but demonstrated rapid ossification after 8 weeks of subcutaneous culture. In comparison, samples from the a7G3P group displayed the highest bone tissue density and degree of mineralization. The degree of ossification increased with an extended subcutaneous culture time in all subgroups.

**Figure 5:**
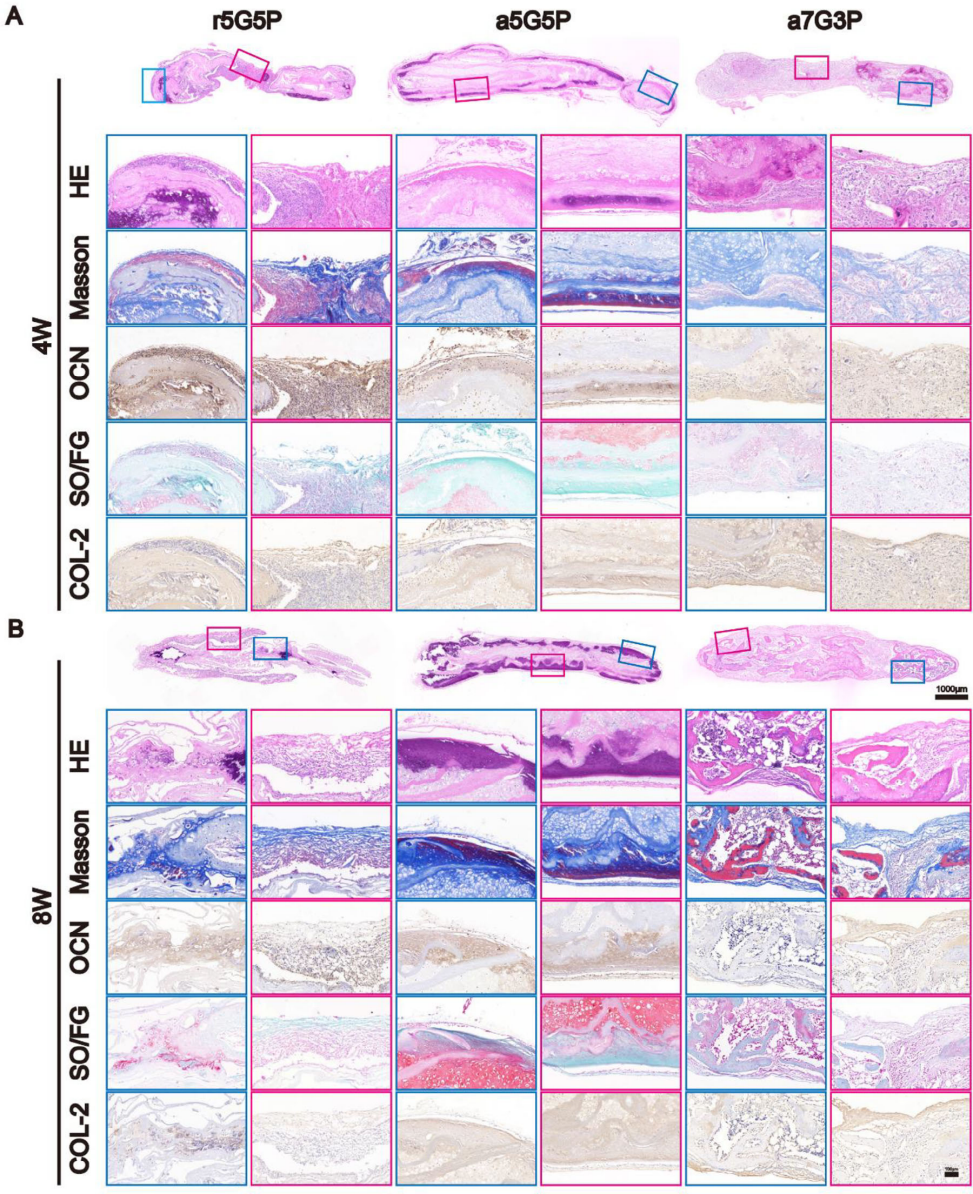
Histological examination of BMSC‐NFMC after subcutaneous implantation for 4 and 8 weeks. A) HE staining, Masson's trichrome staining, OCN staining, SO/FG staining, and COL 2 immunohistochemical staining of BMSC‐NFMC subcutaneous implantation for 4 weeks. B) Corresponding histological examination images after 8 weeks. The red boxes in the figure represent the hybridized tissue regions of the samples, while the blue boxes represent the integrated osteochondral interface regions. Abbreviations: HE, hematoxylin and eosin; OCN, Osteocalcin; SO/FG, Safranin‐O/Fast Green; COL 2, Type II collagen.

Histological analysis revealed that the a5G5P group exhibited the structural characteristics of natural osteochondral integrated tissues (**Figure** **5**; Figure S4A,B, Supporting Information). First, the peripheral layer richly expressed the osteoblast‐related matrix, as verified by Masson staining and positive COL 1 staining, with osteoblasts uniformly distributed, as confirmed by positive ALP and OCN staining. Additionally, the intermediate cartilage layer possessed typical chondrocyte lacunar structures and cartilage‐related extracellular matrix deposition (GAG and COL 2). The boundary structure of the osteochondral tissue is distinct, and no significant fibrous connective tissue formation is observed throughout the regenerated tissue (dual‐color‐boxed area). When the in vivo culture time was extended to 8 weeks, the natural osteochondral integrated structure became more pronounced and stable. In contrast, the other two groups failed to form osteochondral integrated structures: the r5G5P group presented a mixture of disorderly arranged fibrous connective tissue (red boxed area), bone, and cartilage tissue after 4 weeks of in vivo culture. When the in vivo culture time was extended to 8 weeks, a mixture of fibrous connective tissue (red boxed area) and bone tissue was observed. Meanwhile, the a7G3P group showed almost no vascular delay or vascular isolation effects and completely formed bone tissue after 8 weeks of implantation.

These results indicate that the structure and composition of the materials significantly influenced the in vivo tissue regeneration patterns of BMSC‐NFMCs, with the a5G5P group demonstrating the most desirable capacity for osteochondral integrated tissue formation.

### 2.5 Vascular Distribution Characteristics of the BMSC‐NFMC Model and Evaluation of Its Vascular Isolation Effect

**Figure 6:**
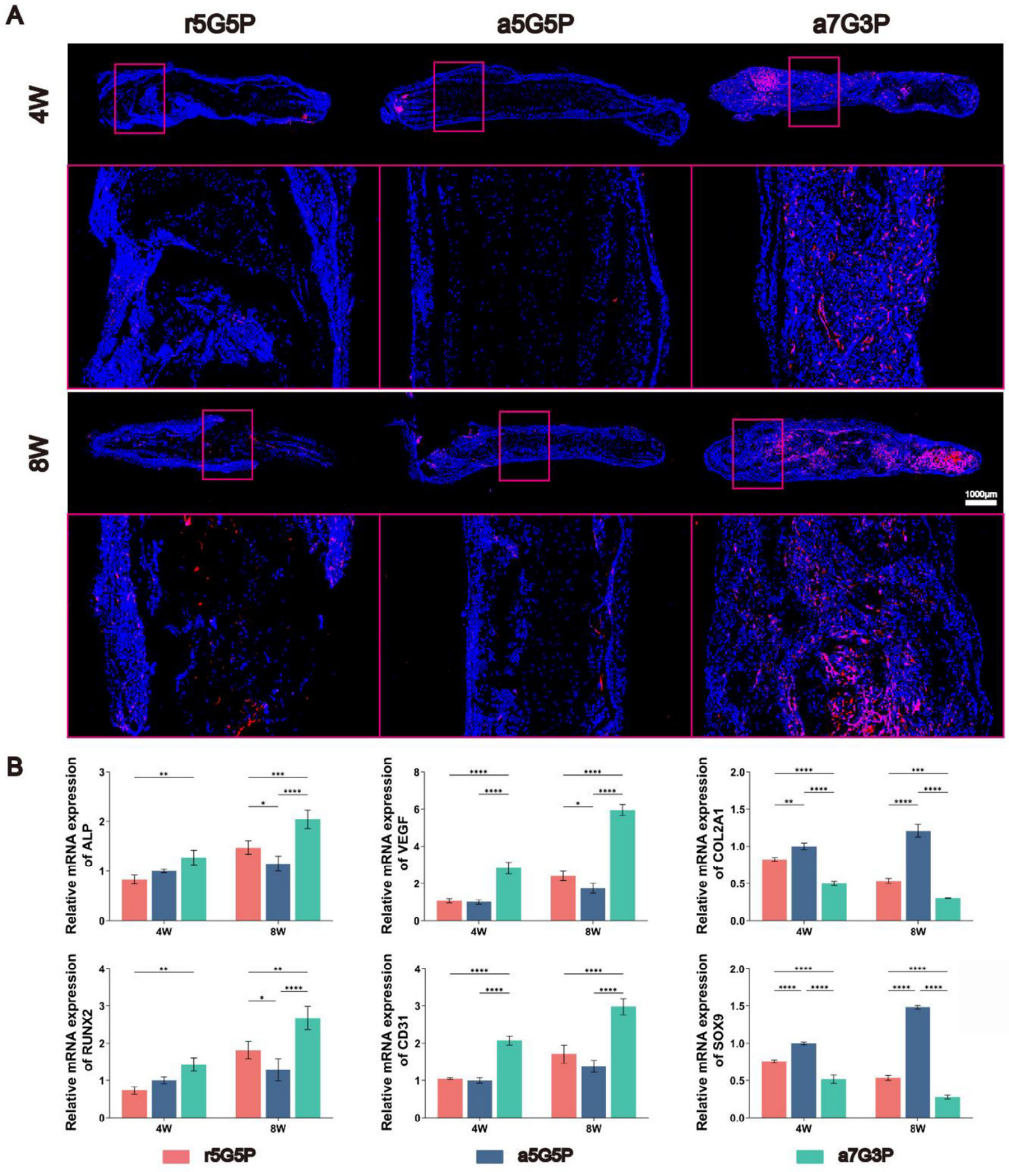
Assessment of vascular distribution and multidirectional differentiation of BMSC‐NFMC after subcutaneous implantation for 4 and 8 weeks. A) Immunofluorescence staining for the specific vascular endothelial cell marker CD31 after 4 weeks and 8 weeks. Endothelial cells are stained red. B) Real‐time polymerase chain reaction (RT‐qPCR) analysis of the expression of osteogenesis‐related genes (*ALP and RUNX2*), angiogenesis‐related genes (*VEGF and CD31*) and chondrogenesis‐related (*SOX9 and COL2A*) via RT‐qPCR in the subcutaneous culture of nude mice for 4 and 8 weeks, with *GADPH* as the reference gene. Data are presented as mean ± SD (*n* = 3). Statistical significance was determined using two‐way ANOVA with Bonferroni's post‐hoc test. ^*^ *p* < 0.05, ^**^ *p* < 0.01, ^***^ *p* < 0.001, ^****^ *p* < 0.0001.

To evaluate the vascular isolation effect of the BMSC‐NFMC model, immunofluorescence staining for the specific vascular endothelial cell marker CD31 was performed in order to analyze the endothelial cells in each group (**Figure** **6**A). Results showed that the a5G5P group exhibited distinct red fluorescence signals in the peripheral region, whereas signals in the central region were sparse. After 8 weeks of subcutaneous culture, this distribution characteristic became more pronounced, with red fluorescence in the peripheral region further intensifying, whereas the central region maintained a relatively low staining intensity, forming a typical spatial gradient distribution. Quantitative analysis of CD31 immunofluorescence images (Figure S4C, Supporting Information) confirmed this observation, revealing a significant vascular density gradient in the a5G5P group. This phenomenon indicates that the a5G5P model could effectively regulate the direction of vascular growth and prevent excessive invasion into the cartilage region. This spatial distribution is important for maintaining a low‐oxygen microenvironment in the cartilage region and promoting natural transitions at the osteochondral interface. In contrast, the r5G5P and a7G3P groups displayed a uniform red fluorescence in both the central and peripheral regions, with no significant differences in vascular density between regions. Additionally, the overall fluorescence signals in the a5G5P and r5G5P groups were relatively weak, while signals in the a7G3P group were the strongest, indicating that the composition and structure of the materials had significant regulatory effects on the degree of vascularization, with the a7G3P group demonstrating the strongest angiogenic capacity.

### 2.6 Assessment of Multidirectional Differentiation Ability of the BMSC‐NFMC Model

The RT‐qPCR results (Figure 6B) demonstrated that at 4 weeks, the expression of angiogenesis‐related genes (*CD31* and *VEGF*) in the a5G5P and r5G5P groups was significantly lower than that in the a7G3P group (*p* < 0.001). By week 8, *VEGF* expression in the a5G5P group was the lowest among all groups, supporting the observations from CD31 immunofluorescence staining.

The RT‐qPCR results (Figure 6B) indicated that at both 4 and 8 weeks, the expression levels of osteogenesis‐related genes (*RUNX2* and *ALP*) in the a7G3P group were significantly higher than those in the other two groups (*p* < 0.01), suggesting that the a7G3P group possessed the strongest osteoinductive potential. Notably, the a5G5P group demonstrated unique advantages in regard to assessing chondrogenic differentiation. When compared to the a7G3P and r5G5P groups, the a5G5P group exhibited significantly higher expression levels of chondrogenesis‐related genes (*COL2A1* and *SOX9*) at both 4 and 8 weeks post implantation (*p* < 0.0001) (Figure 6B). More importantly, the levels of chondrogenic markers (COL2A1 and GAG) in the a5G5P group gradually increased with the extension of the in vivo culture time from four to eight weeks(Figure S3B, Supporting Information), fully demonstrating the excellent ability of the a5G5P group to promote and maintain chondrogenic differentiation.

These gene expression and immunofluorescence analysis results not only mutually validated each other but were also highly aligned with the previous Micro‐CT and histological analysis results, systematically elucidating the unique advantages of different BMSC‐NFMC models in regulating the multidirectional differentiation of BMSCs and vascular growth. In particular, the vascular spatial isolation effect and sustained chondrogenic induction ability exhibited by the a5G5P model provide important experimental evidence for cartilage tissue engineering.

### 2.7 Molecular Mechanisms of Stable Cartilage Formation in the BMSC‐NFMC Model

**Figure 7:**
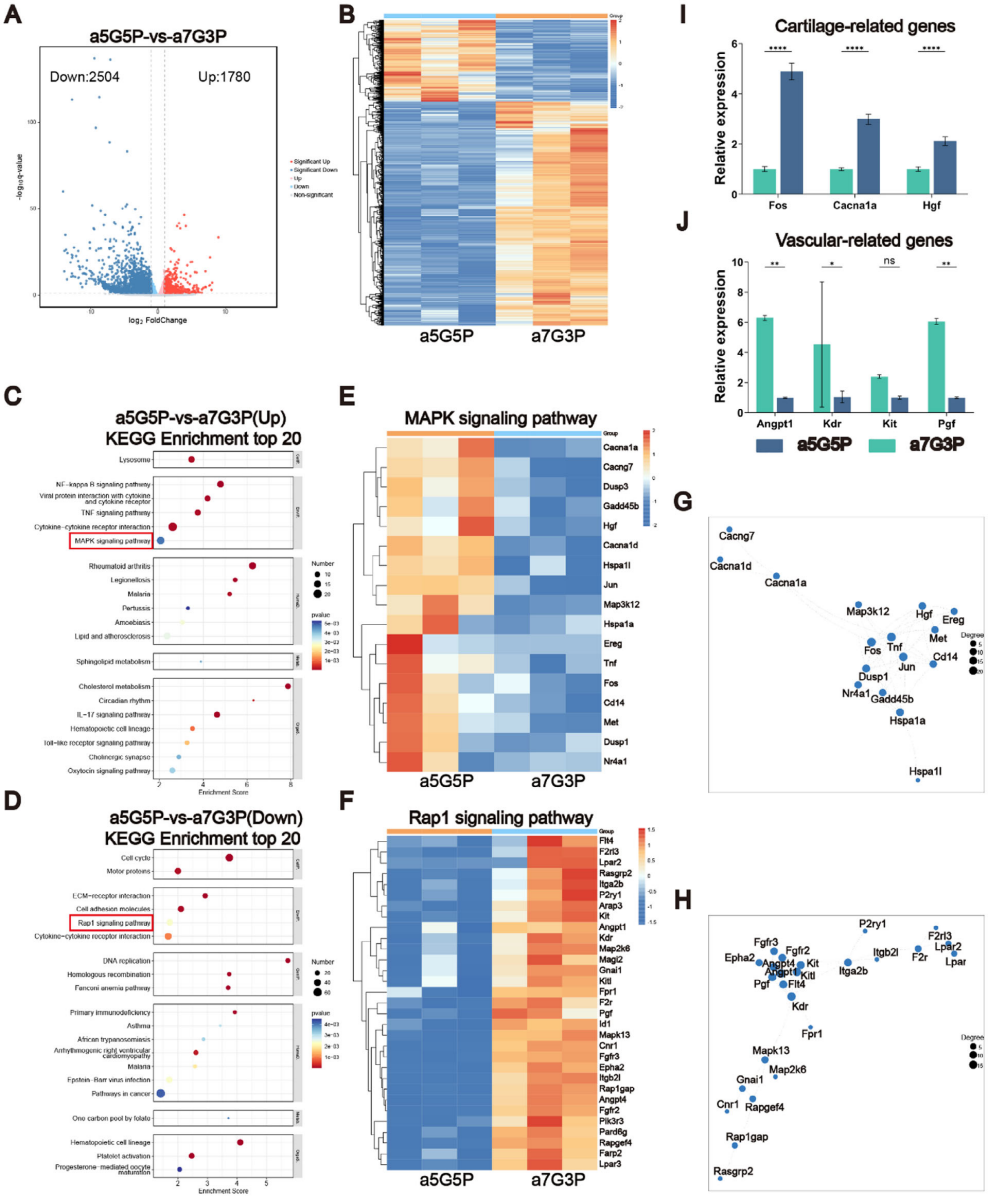
Molecular mechanisms of stable cartilage formation in the BMSC‐NFMC model under ischemic intervention conditions. A,B) Volcano plot and heatmap of differentially expressed genes (DEGs) between the a5G5P and a7G3P groups after 8 weeks of subcutaneous culture in nude mice. C,D) KEGG pathway enrichment analysis of upregulated (C) and downregulated (D) DEGs, with MAPK and Rap1 signaling pathways marked in red, respectively. E,F) Heatmaps of the genes involved in “chondrogenesis regulation” (E) and “angiogenesis regulation” (F). G,H) Gene correlation analysis of biological processes related to “chondrogenesis regulation” (G) and “angiogenesis regulation” (H). I,J) RT‐qPCR validation of cartilage‐related genes *Cacna1a, Hgf*, and *Fos* (I), and angiogenesis‐related genes *Pgf, Kit, Kdr*, and *Angpt1* (J). Data are presented as mean ± SD (*n* = 3 for RNA‐seq analysis; *n* = 3 for RT‐qPCR validation). Statistical significance for gene expression comparisons in I,J was determined using an independent sample *t*‐test. ^*^ *p* < 0.05, ^**^ *p* < 0.01, ^***^ *p* < 0.001, ^****^ *p* < 0.0001, ns: not significant.

To investigate the potential molecular mechanisms of stable cartilage formation in the BMSC‐NFMC model after 8 weeks of subcutaneous culture in nude mice, we conducted genomic analysis of the two groups (a7G3P and a5G5P) to reveal potential gene expression patterns. Transcriptome analysis, visualized through heatmaps and volcano plots, showed significant differentially expressed genes (DEGs) between the two groups: 1780 genes were upregulated and 2504 genes were downregulated in the a5G5P group. This extensive transcriptome remodeling indicated that the a5G5P model successfully constructed a specific microenvironment favorable for cartilage tissue formation and maintenance (**Figure** **7**A,B). The performed KEGG pathway enrichment analysis revealed that differentially expressed genes (DEGs) upregulated in the a5G5P group were primarily enriched in biological processes related to the MAPK signaling pathway (Figure 7C, marked in red). Further gene clustering analysis revealed that these upregulated genes were closely associated with “chondrogenic regulation” and formed a highly correlated gene interaction network (Figure 7E,G). RT‐qPCR confirmed that key cartilage‐related genes, including Cacna1a, Hgf, and Fos, were significantly upregulated (Figure 7I). These genes play important roles in regulating cell proliferation, differentiation, and extracellular matrix synthesis, providing molecular mechanistic support for the robust chondrogenic induction capabilities observed in the a5G5P group. Western blot analysis further verified the activation of the MAPK pathway, with results showing significantly elevated levels of phosphorylated ERK1/2 (p‐ERK1/2) and total ERK1/2 in the experimental group compared to those in the control group. GAPDH, used as a loading control, showed consistent expression in both groups, confirming the reliability of the results (Figure S5B, Supporting Information).

Simultaneously, we observed a significant downregulation of a series of angiogenesis‐related genes in the a5G5P group, particularly key components of the Rap1 signaling pathway (Figure 7D, marked in red). Gene clustering and correlation analyses revealed tight functional connections between the target genes in the Rap1 signaling pathway (Figure 7F,H). Specifically, the expression levels of genes associated with angiogenesis and osteogenesis, such as *Pgf*, *Kit*, *Kdr*, and *Angpt1* were significantly reduced (Figure 7J). These genes primarily participate in the regulation of endothelial cell proliferation, migration, and adhesion, and their suppressed expression further confirmed the characteristic inhibition of angiogenesis at the molecular level in the a5G5P group. Western blot analysis at the protein level showed significantly reduced expression of RAP1A‐GAP and RAP1A in the experimental group, which was consistent with the inhibition of the Rap1 signaling pathway observed in the transcriptome analysis (Figure S5B, Supporting Information).

Based on these aforementioned findings, we propose that the a5G5P model promotes stable cartilage formation through a dual molecular mechanism: on one hand, enhancing the expression of chondrogenic differentiation‐related genes by activating the MAPK signaling pathway, and on the other hand, limiting angiogenesis by inhibiting the Rap1 signaling pathway.

### 2.8 Assessment of BMSC‐NFMC Model Application in Rabbit Articular Osteochondral Defect Repair

To evaluate the potential clinical application of the BMSC‐NFMC model for osteochondral defect repair, we established a standardized cylindrical osteochondral defect model (4 mm in diameter, 4 mm in depth) in rabbit knee joints. a5G5P‐BMSC‐NFMC and a7G3P‐BMSC‐NFMC cultured in vitro for 7 days were implanted into the defect sites, and a blank control group without material implantation was established (Figure S6A, Supporting Information).

**Figure 8:**
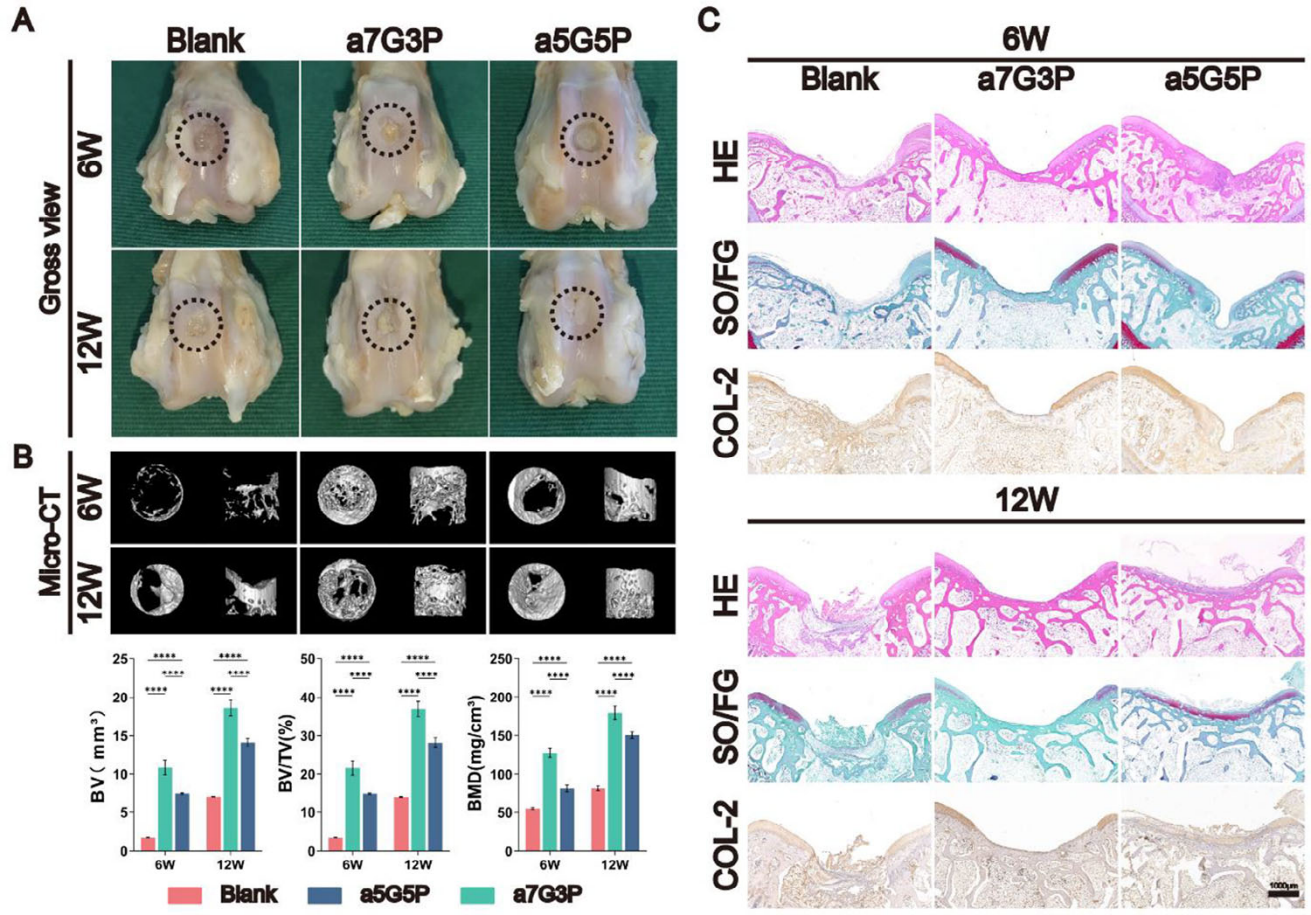
Assessment of BMSC‐NFMC application in rabbit articular osteochondral defect repair. Evaluation of the effectiveness of the BMSC‐NFMC model in repairing rabbit knee joint osteochondral defects including: A) macroscopic observation, B) 3D Micro‐CT reconstruction with quantitative analysis of bone volume (BV), bone surface (BS), bone volume fraction (BV/TV), and bone mineral density (BMD), and C) histological evaluation (H&E, SO/FG, and COL‐2 immunohistochemical staining) of the regenerated osteochondral tissue at 6 and 12 weeks post‐implantation. Data are presented as mean ± SD (*n* = 3 per group). Statistical significance was determined using two‐way ANOVA with Bonferroni's post‐hoc test. Abbreviations: HE, hematoxylin and eosin; SO/FG, Safranin‐O/Fast Green; COL 2, Type II collagen. ^*^ *p* < 0.05, ^**^ *p* < 0.01, ^***^ *p* < 0.001, ^****^ *p* < 0.0001.

In order to comprehensively assess the effectiveness and long‐term stability of the BMSC‐NFMC model in repairing osteochondral defects, samples were collected and analyzed at 6 and 12 weeks post‐implantation. Macroscopic observations showed that at six weeks post‐implantation, the a5G5P group had a smooth, translucent cartilage‐like surface. After 12 weeks, the surface of the defect was almost completely covered with cartilage‐like tissue. In contrast, the a7G3P and blank groups formed yellow tissues with an uneven texture and color on the defect surface (**Figure** **8**A).

Micro‐CT analysis demonstrated that the osteogenic effect was ranked as follows: a7G3P > a5G5P > the blank group. In the blank group, obvious cavities remained in the defect area, whereas the a7G3P and a5G5P groups showed substantial new bone formation, filling the defects, and demonstrating robust osteogenic potential. Statistical analysis further supported the Micro‐CT results, thus confirming the higher bone volume (BV), relative bone volume (BV/TV), and bone mineral density (BMD) (Figure 8B).

The histological evaluation results provided comprehensive evidence for the clinical application of these materials. At both 6 and 12 week time points, the a5G5P group exhibited the most desirable histological characteristics: typical chondrocyte lacuna structures formed in the surface layer with strong positive COL‐2 immunohistochemical staining, SO/FG staining showing rich glycosaminoglycan (GAG) content, and regular subchondral bone formation in the deep layers. In contrast, the blank and a7G3P groups primarily formed fibrous scar tissue with varying degrees of new bone formation; however, no typical hyaline cartilage structures were observed in the surface layer (Figure 8C; Figure S5B, Supporting Information).

These results have systematically demonstrated the differentiated advantages of the BMSC‐NFMC model in osteochondral defect repair: The a5G5P group achieved tissue structure reconstruction highly similar to natural articular tissue by promoting cartilage regeneration and maintaining the cartilage phenotype, whereas the a7G3P group exhibited a stronger osteogenic capacity. This tissue‐specific repair pattern was highly consistent with the previously observed in vitro differentiation characteristics, providing important experimental evidence for personalized treatment strategies targeting different types of osteochondral defects in clinical applications.

## 3 Discussion

The core challenge in osteochondral tissue engineering is achieving the dual objectives of precise vascularization regulation and effective interface integration.^[^ ^42^, ^43^ ^]^ Existing tissue engineering strategies have limitations: single‐layer material structures are simple to prepare but struggle to meet the complex requirements of the osteochondral interface; bi‐layer gradient structures can mimic interface transitions but lack sufficient bonding strength; and multilayer composite structures achieve functional zoning but have significant limitations in vascularization control.^[^ ^44^ ^]^ In this study, we systematically compared the physicochemical characteristics and biological properties of three different materials (r5G5P, a5G5P, and a7G3P) and optimized the GT/PCL ratio and topological structure of nanofibrous materials in order to successfully regulate BMSC differentiation. We innovatively employed the “rolling and folding” method to construct BMSC‐NFMC composite structures, which created a dense physical barrier that achieved effective vascular isolation between bone and cartilage layers. After implantation was performed in nude mice, the a5G5P group (aligned fibers with 50% gelatin/50% PCL) exhibited distinct characteristics of natural osteochondral tissue structure: a superficial cartilage layer of uniform thickness with orderly arranged cells, a clear interface transition zone in the middle, and uniformly distributed osteoblasts in the deep layer. This integrated osteochondral structure became more stable after 8 weeks of incubation in vivo. Transcriptome sequencing analysis of nude mouse samples (a5G5P and a7G3P groups) cultured subcutaneously for eight weeks revealed the following molecular mechanisms under ischemic intervention conditions: inhibition of the Rap1 signaling pathway reduced RAP1A and RAP1A‐GAP protein levels, leading to further activation of the ERK pathway and enhanced ERK1/2 phosphorylation and total protein levels, thereby promoting cartilage‐related gene expression and inhibiting angiogenesis. In the rabbit articular osteochondral defect repair experiments, we evaluated both 6 and 12 week time points, and the results showed that the a5G5P group successfully regenerated complete articular osteochondral structures similar to the adjacent natural tissues. This series of experiments validated the effectiveness of the proposed method.

To address the issue of interface integration, we proposed a strategy to simultaneously construct complete osteochondral structures within the same scaffold. By optimizing the fiber orientation structure and composition ratio, we were able to develop nanofibrous materials capable of supporting tissue‐specific regeneration.^[^ ^45^ ^]^ We compared the physicochemical characteristics of three different materials (r5G5P, a5G5P, and a7G3P), with experimental results showing that the a5G5P combination (aligned fibers with 50% gelatin/50% PCL) demonstrated optimal performance in interface integration: the aligned orientation structure regulated stem cell morphology and arrangement through contact guidance effects^[^ ^41^ ^]^; this proportion of PCL content provided the material with appropriate mechanical strength (Young's modulus ≈8 MPa, tensile strength ≈4 MPa); simultaneously, the presence of gelatin significantly improved the hydrophilicity of the material surface (lower contact angle) and cell adhesion capability, manifested as ≈90% cell adhesion rate and uniform cell distribution. In contrast, the r5G5P group had the same composition ratio but lacked an aligned orientation structure, whereas the a7G3P group had an orientation structure but an excessive gelatin content (70%), which affected its mechanical properties.

This study has addressed a key challenge in osteochondral tissue engineering by developing an innovative BMSC‐NFMC model that achieves integrated osteochondral regeneration. We first constructed BMSC‐NFM using nanofibrous materials with different GT/PCL ratios and surface morphologies, cultured them in vitro for 7 days, then constructed BMSC‐NFMC using the “rolling and folding” method and implanted them in nude mice for evaluation. This method involves “rolling and folding” the cell‐attached nanofibrous membrane in a specific direction, forming a dense physical barrier structure that effectively limits vascular penetration from the periphery to the interior, thereby creating a low‐oxygen environment suitable for chondrogenesis in the interior.^[^ ^46^, ^47^, ^48^, ^49^ ^]^ When compared to other construction approaches for osteochondral interface engineering, our “rolling and folding” method offers several advantages over direct stacking techniques, which often suffer from poor layer integration, chemical crosslinking methods that may introduce cytotoxicity concerns, and 3D printing approaches that generally lack the nanofibrous architecture crucial for cell‐material interactions.^[^ ^50^ ^]^ The experimental results showed that the regenerated tissue in the a5G5P group exhibited ideal osteochondral integration characteristics, including uniform cartilage layer thickness, orderly arranged chondrocytes, obvious cartilage‐related ECM deposition, regular bone layer morphology, uniform osteoblast distribution, and a clear interface structure at the cartilage‐bone tissue junction. As the in vivo culture was extended to 8 weeks, this integrated osteochondral structure became more stable. In contrast, the r5G5P group showed a disorderly tissue arrangement at four weeks and formed a mixed structure of fibrous connective tissue and bone tissue at eight weeks, whereas the a7G3P group lacked effective vascular isolation and eventually completely transformed into bone tissue. These results reveal the key mechanisms by which the BMSC‐NFMC model successfully achieved integrated osteochondral regeneration. First, the experimental results indicated that aligned fibrous membranes exhibited excellent hydrophilicity, directly affecting initial cell adhesion behavior. Observations revealed that the aligned fiber structure not only provided physical guidance for cell growth, but its regular arrangement also significantly improved the wettability of the material surface, facilitating the adsorption of extracellular matrix proteins. This was verified by cell adhesion experiments, which showed higher cell adhesion rates and more uniform cell distribution. Second, the gelatin component in the optimized nanofibrous scaffolds significantly influenced the differentiation direction of BMSCs. The experimental results showed that the a7G3P group (70% gelatin content) significantly upregulated the expression of osteogenic‐related transcription factors *RUNX2* and *ALP*, while also promoting the secretion of angiogenic factors *VEGF* and *CD31*, consistent with its eventual complete transformation into bone tissue. This gelatin content‐dependent regulatory effect acted synergistically with the physical characteristics of the material to jointly regulate the directional differentiation of the BMSCs. Finally, the ‘'rolling and folding'’ method created a local ischemic environment conducive to cartilage regeneration by enhancing the vascular isolation effect, forming a stable physical barrier with the stacked nanofibers as the peripheral bone tissue matured. This method provides an effective technical path for obtaining integrated biomimetic osteochondral structures.

As aforementioned, we successfully constructed subcutaneous biomimetic osteochondral integrated structures in nude mice using the BMSC‐NFMC model. To understand the molecular mechanisms by which the a5G5P‐BMSC‐NFMC scaffold promotes stable cartilage formation, we performed transcriptome sequencing analysis on the a7G3P and a5G5P group samples after 8 weeks of in vivo culture. Sequencing results showed 1780 upregulated and 2504 downregulated genes in the a5G5P group. Cartilage‐related genes (*Cacna1a*, *Hgf*, and *Fos*) and key cartilage markers (*SOX9* and *COL2*) were significantly upregulated in a5G5P group. Through molecular mechanism analysis, we found that enhanced cartilage formation was mainly regulated by the MAPK signaling pathway. Western blotting revealed significantly elevated levels of ERK1/2 phosphorylation and total ERK1/2 expression under ischemic conditions. Based on our results, combined with previous literature reports, among the three major MAPK subfamilies, JNK, p38, and ERK, the ERK pathway (especially ERK2) is significantly activated as a key mediator of chondrogenesis.^[^ ^2^ ^]^ Simultaneously, we observed significant downregulation of angiogenesis‐related genes (*Rap1gap*, *Kit*, *Kdr*, and *Angpt1*), which typically participate in endothelial cell proliferation, migration, and new vessel formation. This result was consistent with the qualitative and quantitative analyses of CD31, confirming that the central cartilage region of the a5G5P group maintained lower vascularization levels during 4 and 8 weeks of culture. Furthermore, Western blot analysis verified changes in the Rap1 signaling pathway, observing dynamic changes in RAP1A and RAP1A‐GAP protein levels under different blood supply conditions, confirming inhibition of the Rap1 pathway. Notably, these two signaling pathways exhibit cross‐regulatory relationships, and research in the literature indicates that Rap1 pathway inhibition can enhance ERK pathway activity, and that the ERK pathway is essential for chondrocyte generation.^[^ ^51^, ^52^, ^53^ ^]^ Therefore, Rap1 inhibition observed in the a5G5P model not only directly limited angiogenesis, but also indirectly promoted chondrogenic differentiation by inhibiting the ERK pathway. This finely tuned molecular regulatory network enables the a5G5P model to construct and maintain a low‐vascularization microenvironment suitable for cartilage development, thereby achieving stable cartilage tissue formation.

Finally, in this study wehave further validated the in situ repair capability of BMSC‐NFMCs in a rabbit knee‐joint defect model. Results showed that the a5G5P group exhibited excellent repair effects at both 6 and 12 weeks post‐implantation, completely achieving structural and functional reconstruction of the defect area by 12 weeks: typical chondrocyte lacunae structures rich in COL‐2 and GAG formed in the surface layer, while regular bone tissue developed in the deep layer. Micro‐CT analysis showed that the osteogenic effect ranking as a7G3P > a5G5P > blank group, confirming the promotion of bone formation by the materials. In contrast, the blank and a7G3P groups primarily formed fibrous scar tissue with varying degrees of new bone generation; however, no typical hyaline cartilage structures were observed on the surface layer. The layered integrated repair pattern achieved by the a5G5P group was similar to that of the natural articular tissue structure, confirming the clinical translation potential of this strategy. When compared with conventional clinical treatments, our BMSC‐NFMC approach offers notable advantages by requiring only a single surgical procedure, generating more natural hyaline cartilage, and simultaneously achieving bone and cartilage regeneration with seamless interface integration. Nevertheless, several key scientific questions still need to be explored in depth on the path from basic research to clinical application.^[^ ^54^, ^55^ ^]^ The precise control mechanisms of three‐dimensional structures and the molecular regulatory network of osteochondral interface formation require further elucidation. Understanding the microenvironmental changes during material degradation and the regulatory effects of degradation products on cell differentiation is particularly important for optimizing material design. Further studies investigating the effects of varying BMSC concentrations on regenerative outcomes will provide additional insights into the optimization of this system for clinical applications. In clinical translation, although our rabbit model provided promising evidence for the efficacy of this approach, significant challenges remain in scaling it up to larger animal models or human applications. The primary task is to validate the long‐term effects of the materials using large animal models, especially by evaluating the sustained impact of different GT/PCL ratios on tissue reconstruction under increased biomechanical loads that more closely mimic human joint conditions. Additionally, validation studies with larger sample sizes are required in order to ensure the reliability and universality of the results. Quality control strategies for material scaled‐up production, evaluation of therapeutic effects under different pathological conditions (such as osteoarthritis and traumatic joint injury), and principles for developing individualized treatment plans also require systematic solutions.

## 4 Conclusion

This study has addressed the core challenge in osteochondral tissue engineering—precise vascularization regulation and interface integration—by systematically comparing both the physicochemical characteristics and biological properties of three different materials (r5G5P, a5G5P, and a7G3P) and developing an osteochondral composite structure based on BMSC‐NFMCs. By optimizing the GT/PCL ratio (50%:50% in a5G5P) and topological structure (aligned fiber orientation) of nanofibrous materials, and employing the “rolling and folding” method to construct BMSC‐NFMC composite structures, effective vascular isolation between the bone and cartilage layers was successfully achieved. Transcriptome sequencing and western blot analysis revealed that under ischemic microenvironmental conditions, the a5G5P group effectively regulated the expression of cartilage‐related genes through inhibition of the Rap1 pathway and subsequent activation of the ERK pathway. Animal experiments demonstrated that the a5G5P group formed stable osteochondral integrated structures subcutaneously in nude mice and successfully regenerated layered tissues similar to natural tissues in rabbit articular defect repair, confirming the effectiveness of this strategy. This approach can not only be used for repairing local articular osteochondral defects but also applied to the repair of long‐segment osteochondral defects in subcutaneous microenvironments (such as finger middle‐segment osteochondral or costal cartilage regeneration), showing broad prospects for clinical application.

## 5 Experimental Section

### Animal Information

In this study, a total of 24 nude mice (12 males and 12 females, 6 weeks old) were purchased from Shanghai Slaccas Experimental Animal Ltd., and 15 New Zealand white rabbits (seven males and eight females, four months old), weighing 2 kg, were purchased from Shanghai Jiagan Biological Technology Co. These animal models were selected because 6‐week‐old nude mice are in a rapid growth and development stage, possess strong tissue regeneration capabilities, and their immunodeficient characteristics are favorable for xenogeneic cell transplantation research; 4‐month‐old New Zealand white rabbits have relatively mature skeletal and cartilage development, with joint anatomical structures similar to humans, thus making them particularly suitable for osteochondral repair model studies. All of the experimental protocols involving animals were approved by the Animal Care and Experimental Committee of the Shanghai Jiao Tong University School of Medicine (approval number SH9H‐2021‐A655‐SB).

### Preparation of Electrospun Nanofibrous Membranes with Different GT/PCL Ratios and Surface Morphologies

Following the aforementioned method, 16% (w/v) electrospun solutions were prepared. Two GT/PCL ratios, 5:5 and 7:3, were selected based on preliminary experiments and literature review, as these two formulations have demonstrated significant potential in terms of mechanical properties, biocompatibility, and degradation characteristics.^[^ ^56^ ^]^ Solutions were prepared by mixing gelatin (GT, Solarbio Biotech Co., China) and polycaprolactone (PCL, M_w_ = 80 000, Perstorp, Sweden) in weight ratios of 5:5 and 7:3, respectively, and dissolving them in hexafluoroisopropanol (HFIP, Sigma, USA). After dissolution, the two GT/PCL solutions were loaded into 10 mL syringes equipped with 25G blunt‐tip needles for electrospinning. The electrospinning parameters were as follows: feed rate: 0.3 mm min^−1^, collection distance: 10 cm; and voltage: 11 kV. Additionally, aligned and randomly oriented nanofibrous membranes were fabricated by setting the collector rotation speed to 3000 and 100 rpm, respectively. Three types of nanofibrous membranes were prepared: r5G5P, a5G5P, and a7G3P. Following electrospinning, the nanofibrous membranes were dried under vacuum and chemically crosslinked. Subsequently, the membranes were sterilized in 75% ethanol for 30 min, washed thrice with PBS for 5 min each, and then sterilized under UV light for 30 min on each side.^[^ ^57^ ^]^

### Characterization of Nanofibrous Membranes

The morphologies of the three types of nanofibrous membranes were observed using SEM (Zeiss GeminiSEM 300, Germany).^[^ ^58^ ^]^ The diameters of 40–50 individual nanofibers in each group were measured using ImageJ software.

The mechanical properties of the scaffolds (*n* = 4 per group) were analyzed in the wet state using a biomechanical testing machine (INSTRON 3367, USA). Rectangular samples (20 mm × 10 mm) were stretched at a constant crosshead speed of 10 mm min^−1^. The tensile strength, Young's modulus, and elongation at break for all groups were determined based on the stress–strain curves.^[^ ^56^ ^]^

The hydrophilicity of the scaffolds was assessed as described previously.^[^ ^59^ ^]^ Deionized water (0.5 mL) was dropped automatically onto the surface of the flat GT/PCL membrane. The contact angle, which is indicative of the material's wettability, was measured and calculated using a contact angle/surface tension measurement instrument (Dataphysics OCA20, Germany).

### Isolation and Culture of BMSCs

Bone marrow was aspirated from the anterior superior iliac spine of healthy New Zealand rabbits. The BMSCs were isolated and cultured in a standard culture medium composed of low‐glucose Dulbecco's modified Eagle's medium (DMEM; Gibco BRL, Grand Island, NY, USA) supplemented with 10% fetal bovine serum (FBS; Hyclone, Logan, UT, USA), following previously established methods. BMSCs at the second passage (P2) were used for the subsequent experiments.^[^ ^60^ ^]^

### Cell adhesion and cell seeding efficiency of Nanofibrous Membranes

Nanofibrous membranes were cut into circles with a diameter of 1.5 cm and then placed in 24‐well plates. A suspension of collected BMSCs (1.0 × 10⁵ cells mL^−1^) was evenly dripped onto the three groups of membranes separately. The samples were then incubated to allow full adhesion of the BMSCs to the scaffolds. After 24 h of incubation, the samples were gently transferred to new 6‐well plates and fixed overnight in 0.05% glutaraldehyde at 4°C. Cell adhesion was examined by SEM following dehydration and critical‐point drying. Culture media from each group were collected at the end of the incubation, and the number of unattached (lost) cells was counted. The cell‐seeding efficiency of the samples was calculated using the following formula: (total cell count–lost cell count) / total cell count × 100%.^[^ ^61^ ^]^

### Cell Viability on Nanofibrous Membranes

A suspension of BMSCs (1.0 × 10⁵ cells mL^−1^) was evenly seeded onto the membranes and incubated at 37°C in a 5% CO₂ incubator. The viability of the BMSCs on the three types of nanofibrous membranes was determined using a Calcein‐AM/PI (live/dead) staining kit (C542, Dojindo, Shanghai, China) after 24 h of culture, following the manufacturer's instructions.

Fluorescence images were obtained using a Thunder Imager 3D Tissue Fluorescence Microscope (SP5; Leica, Germany). Cell proliferation was assessed using the CCK‐8 assay (CK04; Dojindo, Kumamoto, Japan) on days 1, 3, 5, 7, 9, and 11 of culture following the manufacturer's instructions.^[^ ^38^ ^]^

### Cell Morphology on Nanofibrous Membranes

After BMSCs were seeded onto the nanofibrous membranes and cultured for 3 days, they were fixed with 4% paraformaldehyde for 30 min. The cells were then permeabilized for 10 minutes in PBS solution containing 500 µL of 0.2% (v/v) Triton X‐100 (Aldrich, USA). Subsequently, cytoskeletal protein (F‐actin) (CA1620, Solarbio, Shanghai, China) was incubated with the cells for 30 min at room temperature in the dark, and the nuclei were stained with DAPI (C1006, Beyotime, Shanghai, China). The morphology of BMSCs on the three types of nanofibrous membranes was observed using an inverted fluorescence microscope, and cell alignment and morphological characteristics were further analyzed using ImageJ software.^[^ ^62^ ^]^

### Construction of BMSC‐NFMCs

The construction of the BMSC‐NFMCs followed a strictly standardized approach. The three types of nanofibrous membranes (10 mm × 10 mm) were placed in culture dishes, and BMSCs suspension (100 µL, cell density 1.0 × 10⁸ cells mL^−1^) was seeded using a precision micropipette. To ensure consistency between batches, cell suspension preparation and seeding were performed by the same operator using standardized cell counting and dilution methods. Three types of BMSC‐nanofibrous NFMs were constructed: r5G5P‐BMSC‐NFM, a5G5P‐BMSC‐NFM, and a7G3P‐BMSC‐NFM. After 4 h of cell incubation, the culture medium (low‐glucose DMEM containing 10% FBS) was gently added at a standardized rate to cover the BMSC‐NFMs. Cultures were maintained at 37°C, 95% humidity, and 5% CO₂, with medium changes every three days. After 7 days of incubation, “rolling and folding” were performed under aseptic conditions using dedicated pressure plates and tweezers with strictly controlled parameters. Specifically, the BMSC‐NFM was rolled longitudinally from one end to the other with uniform pressure (2.0 ± 0.2 kPa) applied perpendicular (90°) to the membrane surface. The entire procedure was completed at room temperature (25°C) within 2 min to minimize cell exposure to nonculture conditions. This process resulted in the formation of BMSC‐NFMCs composed of four layers of nanofibrous membranes. Three constructs were prepared: r5G5P‐BMSC‐NFMC, a5G5P‐BMSC‐NFMC, and a7G3P‐BMSC‐NFMC. Finally, HE staining was performed on samples from each group during the preparation for implantation.^[^ ^63^ ^]^

### Subcutaneous Implantation in Nude Mice

The Engineered BMSC‐NFMCs were subcutaneously implanted into the dorsal flanks of nude mice (*n* = 8 per group). The mice were maintained for 4 and 8 weeks, at which time all in vivo samples were harvested to evaluate bone‐cartilage integration and regeneration.

### Osteochondral Defect Repair in Rabbits

The a5G5P‐BMSC‐NFMC, and a7G3P‐BMSC‐NFMC were cultured in vitro for 7 days and prepared into cylindrical blocks measuring 4 mm in diameter and 4 mm in thickness using a ring drill. Following anesthesia, preoperative skin preparation, and disinfection, a medial parapatellar incision was made, and arthrotomy of the knee joint was performed, thus resulting in lateral patellar dislocation. A cylindrical osteochondral defect measuring 4 mm in diameter and 4 mm in depth was created in the distal femoral trochlear groove using a corneal ring drill. After removing the thrombus, the a5G5P‐BMSC‐NFMC and a7G3P‐BMSC‐NFMC were implanted into the defects, while the remaining defects served as the blank control group (*n* = 4 rabbits). The constructs were secured by suturing the surrounding natural cartilage with biodegradable sutures.

### Samples Harvested

Regenerated tissues were collected after 4 and 8 weeks of subcutaneous implantation in nude mice and after 6 and 12 weeks from rabbit joint defect sites. After gross images were captured, the tissues were fixed in 4% PFA for subsequent analysis.

### Micro‐computed Tomography (Micro‐CT) Scanning

The Harvested samples of nude mice and rabbits were scanned by micro‐CT (VENUS Micro‐CT VNC‐102, Pingsheng Medical, Kunshan, China) to obtain three‐dimensional reconstructions. Furthermore, data obtained by micro‐CT analyses were used to calculate bone volume (BV; mm^3^), bone volume fraction (BV/TV), bone surface area (BS; mm^2^) and trabecular mineral density (TMD).

### Histological and Immunohistochemical Analysis

To evaluate the feasibility of the simultaneous construction of bone‐cartilage integrated tissue in this study, all harvested engineered tissues were subjected to histological and immunohistochemical examinations after decalcification, dehydration, paraffin embedding, and sectioning. HE, safranin‐O/fast green (SO/FG), and immunohistochemical staining for type II collagen were performed to observe the histological structure and cartilage matrix deposition of GAG and COL 2, respectively. Immunohistochemical analyses of COL 1 and Masson's trichrome staining were performed to evaluate bone collagen expression.

The expression of osteoblasts in the samples was assessed using an alkaline phosphatase (ALP) assay kit (1:200 in PBS, pa5‐69994, Invitrogen), as previously described. Subsequently, a rabbit polyclonal antibody against OCN (1:50 in PBS, df12303, Affinity) was used to detect the expression of OCN (a marker protein of late‐stage osteoblast differentiation) in accordance with the manufacturer's instructions.

Thereafter, CD31 immunofluorescence staining was conducted using an anti‐CD31 antibody (1:50, ab28364, Abcam) to observe angiogenesis in the subcutaneous regenerated tissue in nude mice.

### Biochemical Analysis

The glycosaminoglycan (GAG) content of the engineered tissues was quantified at 4 and 8 weeks using the dimethylmethylene blue (DMMB) method (Sigma, Aldrich), as previously described.^[^ ^64^ ^]^ Additionally, the type II collagen content in the engineered tissues was quantified using ELISA.^[^ ^65^ ^]^

### Real‐time Quantitative Polymerase Chain Reaction (RT‐qPCR) Analysis

RT‐qPCR was performed in order to quantify the expression of osteogenesis‐related genes (*RUNX2, ALP*), chondrogenesis‐related genes (*SOX9, COL2A1*), and angiogenesis‐related genes *(CD31, VEGF*) using *GAPDH* as the reference gene for all BMSC‐NFMCs constructed in vitro and for nude mouse subcutaneous tissue regeneration. All tests were performed in triplicate, normalized relative to the expression of housekeeping gene *GADPH*, and analyzed using the 2− ΔCt method. Primer sequences are provided in Table S1 (Supporting Information).

### Transcriptome Sequencing Analysis of Eukaryotic Parameters

The a5G5P‐BMSC‐NFMC and a7G3P‐BMSC‐NFMC were collected under aseptic conditions after 8 weeks of subcutaneous culture in nude mice for transcriptome sequencing analysis of eukaryotic parameters. Briefly, total RNA was extracted using the TRIzol reagent (Invitrogen, Carlsbad, CA, USA) following the manufacturer's protocol. The RNA purity and concentration were assessed using the NanoDrop 2000 spectrophotometer (Thermo Scientific, USA), while RNA integrity was evaluated with the Agilent 2100 Bioanalyzer (Agilent Technologies, Santa Clara, CA, USA). Subsequently, libraries were prepared using the VAHTS Universal V6 RNA‐seq Library Prep Kit according to the manufacturer's instructions. Sequencing was performed on an Illumina NovaSeq 6000 platform, generating 150 bp paired‐end reads. Differential expression analysis was conducted using DESeq2. All transcriptome sequencing and analyses were performed by OE Biotech Co. Ltd. (Shanghai, China).

### Statistical Analysis

Data were checked for normality using Shapiro–Wilk test and for outliers using Grubbs' test (*α* = 0.05). All data are presented as mean ± SD. One‐way ANOVA with Tukey's post‐hoc test was used for single‐variable comparisons across multiple groups. An independent sample *t*‐test was used to compare two groups. Paired t‐tests were used to compare matched samples within the same group (e.g., central vs. peripheral regions of the same construct). Two‐way ANOVA with Bonferroni's post‐hoc test was used to analyze two variables (treatment group and time point). The homogeneity of variance was verified using Levene's test. All tests were two‐sided, with significance set at *p* < 0.05. ^**^Significance levels were defined as follows: ^*^ *p* < 0.05, ^**^ *p* < 0.01, ^***^ *p* < 0.001, ^**^ *p* < 0.0001, and ns (not significant). Statistical analyses were performed using GraphPad Prism 8.0 (GraphPad Software).

## Conflict of Interest

The authors declare no conflict of interest.

## Supporting information



Supporting Information

## Data Availability

The data that support the findings of this study are available from the corresponding author upon reasonable request.

## References

[1] Z. Fang , G. Liu , B. Wang , H. Meng , A. Bahatibieke , J. Li , M. Ma , J. Peng , Y. Zheng , Carbohydr. Polym. 2024, 343, 122424. 39174114 10.1016/j.carbpol.2024.122424

[2] S.‐J. Wang , D. Jiang , Z.‐Z. Zhang , Y.‐R. Chen , Z.‐D. Yang , J.‐Y. Zhang , J. Shi , X. Wang , J.‐K. Yu , Adv. Mater. 2019, 31, 1904341. 10.1002/adma.201904341 31621958

[3] G. Yang , H. Lin , B. B. Rothrauff , S. Yu , R. S. Tuan , Acta Biomater. 2016, 35, 68. 26945631 10.1016/j.actbio.2016.03.004 PMC5408748

[4] A. Baawad , D. Jacho , T. Hamil , E. Yildirim‐Ayan , D.‐S. Kim , Tissue Eng., Part B 2023, 29, 123. 10.1089/ten.TEB.2022.0114 36181352

[5] R. Chen , J. S. Pye , J. Li , C. B. Little , J. J. Li , Bioact. Mater. 2023, 27, 505. 37180643 10.1016/j.bioactmat.2023.04.016 PMC10173014

[6] T.‐Y. Chen , N.‐T. Dai , T.‐K. Wen , S.‐H. Hsu , Adv. Healthcare Mater. 2024, 13, 2400462. 10.1002/adhm.202400462 38948966

[7] C. Deng , J. Chang , C. Wu , J. Orthop. Translation 2019, 17, 15. 10.1016/j.jot.2018.11.006 PMC6551354 31194079

[8] W. He , C. Li , S. Zhao , Z. Li , J. Wu , J. Li , H. Zhou , Y. Yand , Y. Xu , H. Xia , Bioact. Mater. 2024, 34, 338. 38274295 10.1016/j.bioactmat.2023.12.020 PMC10809007

[9] S. Ansari , S. Khorshidi , A. Karkhaneh , Acta Biomater. 2019, 87, 41. 30721785 10.1016/j.actbio.2019.01.071

[10] M. Kazemi , J. L. Williams , Cartilage 2021, 13, 16S. 32458695 10.1177/1947603520924776 PMC8804776

[11] A. P. Kusumbe , S. K. Ramasamy , R. H. Adams , Nature 2014, 507, 323. 24646994 10.1038/nature13145 PMC4943525

[12] X. Wang , Q. Wu , R. Zhang , Z. Fan , W. Li , R. Mao , Z. Du , X. Yao , Y. Ma , Y. Yan , W. Sun , H. Wu , W. Wei , Y. Hu , Y. Hong , H. Hu , Y. W. Koh , W. Duan , X. Chen , H. Ouyang , Ann. Rheum. Dis. 2023, 82, 393. 36261249 10.1136/ard-2022-222944

[13] D. J. Hunter , L. March , M. Chew , Lancet 2020, 396, 1711. 33159851 10.1016/S0140-6736(20)32230-3

[14] C. Vinatier , J. Guicheux , Ann. Phys. Rehabilitation Med. 2016, 59, 139. 10.1016/j.rehab.2016.03.002 27079583

[15] N. Bakhtiary , C. Liu , F. Ghorbani , Gels 2021, 7, 274. 34940334 10.3390/gels7040274 PMC8700778

[16] R. Cao , Y. Xu , Y. Xu , D. D. Brand , G. Zhou , K. Xiao , H. Xia , J. T. Czernuszka , Adv. Healthcare Mater. 2022, 11, 2101643. 10.1002/adhm.202101643 35134274

[17] R. Choe , E. Devoy , B. Kuzemchak , M. Sherry , E. Jabari , J. D. Packer , J. P. Fisher , Biofabrication 2022, 14. 10.1088/1758-5090/ac5220 PMC8918066 35120345

[18] X. Niu , N. Li , Z. Du , X. Li , Bioact Mater 2023, 20, 574. 35846846 10.1016/j.bioactmat.2022.06.011 PMC9254262

[19] S. Wang , S. Zhao , J. Yu , Z. Gu , Y. Zhang , Small 2022, 18, 2201869. 10.1002/smll.202201869 35713246

[20] W. Wei , H. Dai , Bioact. Mater. 2021, 6, 4830. 34136726 10.1016/j.bioactmat.2021.05.011 PMC8175243

[21] S. K. Ramasamy , A. P. Kusumbe , L. Wang , R. H. Adams , Nature 2014, 507, 376. 24647000 10.1038/nature13146 PMC4943529

[22] J. Tuckermann , R. H. Adams , Nat. Rev. Rheumatol. 2021, 17, 608. 34480164 10.1038/s41584-021-00682-3 PMC7612477

[23] Y. J. Hu , Y. E. Yu , H. J. Cooper , R. P. Shah , J. A. Geller , X. L. Lu , E. Shane , J. Bathon , N. E. Lane , X. E. Guo , J. Bone Miner. Res. 2024, 39, 1120. 38887013 10.1093/jbmr/zjae094 PMC12102592

[24] P. Chen , L. Zheng , Y. Wang , M. Tao , Z. Xie , C. Xia , C. Gu , J. Chen , P. Qiu , S. Mei , L. Ning , Y. Shi , C. Fang , S. Fan , X. Lin , Theranostics 2019, 9, 2439. 31131046 10.7150/thno.31017 PMC6525998

[25] X. Ren , F. Wang , C. Chen , X. Gong , L. Yin , L. Yang , BMC Musculoskeletal Disord. 2016, 17, 301. 10.1186/s12891-016-1130-8 PMC4955200 27439428

[26] W. Dai , M. Sun , X. Leng , X. Hu , Y. Ao , Front. Bioeng. Biotechnol. 2020, 8, 604814. 33330436 10.3389/fbioe.2020.604814 PMC7729093

[27] S. Korpayev , G. Kaygusuz , M. Sen , K. Orhan , Ç. Oto , A. Karakeçili , Int. J. Biol. Macromol. 2020, 156, 681. 32320808 10.1016/j.ijbiomac.2020.04.109

[28] B. Zhang , J. Huang , R. J. Narayan , J. Mater. Chem. B 2020, 8, 8149. 32776030 10.1039/d0tb00688b

[29] L. Cheng , L. Gao , W. Guan , J. Mao , W. Hu , B. Qiu , J. Zhao , Y. Yu , G. Pei , Cell Res. 2015, 25, 1269. 26427716 10.1038/cr.2015.120 PMC4650423

[30] G. Yang , Q. He , X. Guo , R.‐Y. Li , J. Lin , Y. Lang , W. Tao , W. Liu , H. Lin , S. Xing , Y. Qi , Z. Xie , J.‐D. J. Han , B. Zhou , Y. Teng , X. Yang , Sci. Adv. 2024, 10, adl2238. 10.1126/sciadv.adl2238 PMC10889359 38394209

[31] M. Y. Hoover , T. H. Ambrosi , H. M. Steininger , L. S. Koepke , Y. Wang , L. Zhao , M. P. Murphy , A. A. Alam , E. J. Arouge , M. G. K. Butler , E. Takematsu , S. P. Stavitsky , S. Hu , D. Sahoo , R. Sinha , M. Morri , N. Neff , J. Bishop , M. Gardner , S. Goodman , M. Longaker , C. K. F. Chan , Nat. Protoc. 2023, 18, 2256. 37316563 10.1038/s41596-023-00836-5 PMC10495180

[32] Y. Hu , Y. Zhang , C.‐Y. Ni , C.‐Y. Chen , S.‐S. Rao , H. Yin , J. Huang , Y.‐J. Tan , Z.‐X. Wang , J. Cao , Z.‐Z. Liu , P.‐L. Xie , B. Wu , J. Luo , H. Xie , Theranostics 2020, 10, 2293. 32089743 10.7150/thno.39238 PMC7019162

[33] A. C. Daly , F. E. Freeman , T. Gonzalez‐Fernandez , S. E. Critchley , J. Nulty , D. J. Kelly , Adv. Healthcare Mater. 2017, 6. 10.1002/adhm.201700298 28804984

[34] G. Huang , F. Li , X. Zhao , Y. Ma , Y. Li , M. Lin , G. Jin , T. J. Lu , G. M. Genin , F. Xu , Chem. Rev. 2017, 117, 12764. 28991456 10.1021/acs.chemrev.7b00094 PMC6494624

[35] Z. Álvarez , J. A. Ortega , K. Sato , I. R. Sasselli , A. N. Kolberg‐Edelbrock , R. Qiu , K. A. Marshall , T. P. Nguyen , C. S. Smith , K. A. Quinlan , V. Papakis , Z. Syrgiannis , N. A. Sather , C. Musumeci , E. Engel , S. I. Stupp , E. Kiskinis , Cell Stem Cell 2023, 30, 219. 36638801 10.1016/j.stem.2022.12.010 PMC9898161

[36] T.‐J. Ji , B. Feng , J. Shen , M. Zhang , Y.‐Q. Hu , A.‐X. Jiang , D.‐Q. Zhu , Y.‐W. Chen , W. Ji , Z. Zhang , H. Zhang , F. Li , Adv. Sci. (Weinh) 2021, 8, 2100351. 34453784 10.1002/advs.202100351 PMC8529489

[37] L. Xiao , H. Liu , H. Huang , S. Wu , L. Xue , Z. Geng , L. Cai , F. Yan , J. Nanobiotechnol. 2024, 22, 322. 10.1186/s12951-024-02578-2 PMC11162076 38849858

[38] X. Zhu , Y. Xu , X. Xu , J. Zhu , L. Chen , Y. Xu , Y. Yang , N. Song , Small 2022, 18, 2201874. 10.1002/smll.202201874 35557029

[39] J. Xue , T. Wu , Y. Dai , Y. Xia , Chem. Rev. 2019, 119, 5298. 30916938 10.1021/acs.chemrev.8b00593 PMC6589095

[40] W. Kitana , V. Levario‐Diaz , E. A. Cavalcanti‐Adam , L. Ionov , Adv. Healthcare Mater. 2024, 13, 2303343. 10.1002/adhm.202303343 PMC11469018 38009530

[41] M. Rashtchian , A. Hivechi , S. H. Bahrami , P. B. Milan , S. Simorgh , Carbohydr. Polym. 2020, 233, 115873. 32059913 10.1016/j.carbpol.2020.115873

[42] J. I. Kim , J. Y. Kim , C. H. Park , Sci. Rep. 2018, 8, 3424. 29467436 10.1038/s41598-018-21618-0 PMC5821851

[43] J. Zhang , L. Liao , J. Zhu , X. Wan , M. Xie , H. Zhang , M. Zhang , L. Lu , H. Yang , D. Jing , X. Liu , S. Yu , X. L. Lu , C. Chen , Z. Shan , M. Wang , J. Dental Res. 2018, 97, 563. 10.1177/0022034517748562 29298566

[44] M. Rahmati , E. A. Silva , J. E. Reseland , C. A. Heyward , H. J. Haugen , Chem. Soc. Rev. 2020, 49, 5178. 32642749 10.1039/d0cs00103a

[45] Y. Zhang , J. R. Venugopal , A. El‐Turki , S. Ramakrishna , B. Su , C. T. Lim , Biomaterials 2008, 29, 4314. 18715637 10.1016/j.biomaterials.2008.07.038

[46] N.‐C. Cheng , B. T. Estes , H. A. Awad , F. Guilak , Tissue Eng., Part A 2009, 15, 231. 18950290 10.1089/ten.tea.2008.0253 PMC3592270

[47] G. M. Cunniffe , P. J. Díaz‐Payno , E. J. Sheehy , S. E. Critchley , H. V. Almeida , P. Pitacco , S. F. Carroll , O. R. Mahon , A. Dunne , T. J. Levingstone , C. J. Moran , R. T. Brady , F. J. O'Brien , P. A. J. Brama , D. J. Kelly , Biomaterials 2019, 188, 63. 30321864 10.1016/j.biomaterials.2018.09.044

[48] E. Y. Salinas , J. C. Hu , K. Athanasiou , Tissue Eng., Part B 2018, 24, 345. 10.1089/ten.teb.2018.0006 PMC6199627 29562835

[49] X. Zhou , T. Esworthy , S.‐J. Lee , S. Miao , H. Cui , M. Plesiniak , H. Fenniri , T. Webster , R. D. Rao , L. G. Zhang , Nanomed.: Nanotechnol., Biol. Med. 2019, 19, 58. 10.1016/j.nano.2019.04.002 31004813

[50] A. S. Tiffany , B. A. C. Harley , Adv. Healthcare Mater. 2022, 11, 2200471.

[51] H. Li , T. Zhao , Z. Yuan , T. Gao , Y. Yang , R. Li , Q. Tian , P. Tang , Q. Guo , L. Zhang , Bioact. Mater. 2024, 41, 61. 39104774 10.1016/j.bioactmat.2024.06.037 PMC11299526

[52] G. Tian , H. Yin , J. Zheng , R. Yu , Z. Ding , Z. Yan , Y. Tang , J. Wu , C. Ning , X. Yuan , C. Liao , X. Sui , Z. Zhao , S. Liu , W. Guo , Q. Guo , Bioact. Mater. 2024, 41, 455. 39188379 10.1016/j.bioactmat.2024.07.034 PMC11347043

[53] W. Wang , Q. Wang , S. Sun , P. Zhang , Y. Li , W. Lin , Q. Li , X. Zhang , Z. Ma , H. Lu , Int. J. Oral Sci. 2024, 16, 12. 38311610 10.1038/s41368-023-00272-x PMC10838930

[54] L. Xu , Y. Liu , Y. Sun , B. Wang , Y. Xiong , W. Lin , Q. Wei , H. Wang , W. He , B. Wang , G. Li , Stem Cell Res. Therapy 2017, 8, 275. 10.1186/s13287-017-0716-x PMC5718061 29208029

[55] S. Yin , W. Zhang , Z. Zhang , X. Jiang , Adv. Healthcare Mater. 2019, 8, 1801433. 10.1002/adhm.201801433 30938094

[56] R. Zheng , H. Duan , J. Xue , Y. Liu , B. Feng , S. Zhao , Y. Zhu , Y. Liu , A. He , W. Zhang , W. Liu , Y. Cao , G. Zhou , Biomaterials 2014, 35, 152. 24135269 10.1016/j.biomaterials.2013.09.082

[57] L. Ghasemi‐Mobarakeh , M. P. Prabhakaran , M. Morshed , M.‐H. Nasr‐Esfahani , S. Ramakrishna , Biomaterials 2008, 29, 4532. 18757094 10.1016/j.biomaterials.2008.08.007

[58] R. Zheng , X. Wang , J. Xue , L. Yao , G. Wu , B. Yi , M. Hou , H. Xu , R. Zhang , J. Chen , Z. Shen , Y. Liu , G. Zhou , Front. Bioeng. Biotechnol. 2021, 9, 752677. 34993184 10.3389/fbioe.2021.752677 PMC8724256

[59] C. He , Q. Lv , Z. Liu , S. Long , H. Li , Y. Xiao , X. Yang , Y. Liu , C. Liu , Z. Wang , J. Biomater. Appl. 2023, 37, 1582. 36662630 10.1177/08853282221144220

[60] B. Bai , J. Hao , M. Hou , T. Wang , X. Wu , Y. Liu , Y. Wang , C. Dai , Y. Hua , G. Ji , G. Zhou , ACS Appl. Mater. Interfaces 2022, 14, 42388. 36094886 10.1021/acsami.2c08422

[61] J. Hao , B. Bai , Z. Ci , J. Tang , G. Hu , C. Dai , M. Yu , M. Li , W. Zhang , Y. Zhang , W. Ren , Y. Hua , J. Hao , G. Zhou , Bioact. Mater. 2022, 14, 97 35310359 10.1016/j.bioactmat.2021.12.013 PMC8892219

[62] B. Yi , Y. Shen , H. Tang , X. Wang , B. Li , Y. Zhang , ACS Appl. Mater. Interfaces 2019, 11, 6867. 30676736 10.1021/acsami.9b00293

[63] H. Zhang , Y. Zhou , W. Zhang , K. Wang , L. Xu , H. Ma , Y. Deng , Acta Biomater. 2018, 77, 212. 30017924 10.1016/j.actbio.2018.07.024

[64] Z. Li , R. Ba , Z. Wang , J. Wei , Y. Zhao , W. Wu , Stem Cells Transl. Med. 2017, 6, 601. 28191761 10.5966/sctm.2016-0050 PMC5442805

[65] A. G. Mikos , M. D. Lyman , L. E. Freed , R. Langer , Biomaterials 1994, 15, 55. 8161659 10.1016/0142-9612(94)90197-x

