# Supplementary material for: BMSC‐NFMC Model for Vascular Regulation and Interface Integration in Osteochondral Regeneration
Source: Adv Sci (Weinh). 2025 Jun 23;12(33):e05222. doi: 10.1002/advs.202505222 (PMC12412503; doi:10.1002/advs.202505222)
Supplement: Supplementary file 1 — Supporting Information [file ADVS-12-e05222-s001.docx]

Supporting Information

**BMSC-NFMC Model for Vascular Regulation and Interface Integration in Osteochondral Regeneration**

*Qian Zhou, Mengjie Hou, Baoshuai Bai, Yiwu Zhang, Yiwei Shen, Zenghui Jia, Yongqiang Guo*, Guangdong Zhou*, and Xiaoqin Liang**

**Q. Zhou, Y. Zhang, Y. Shen, Z. Jia, G. Zhou, X. Liang**

Plastic Surgery Institute, Shandong Second Medical University, Weifang, Shandong 261053, PR China

E-mail: G. Zhou: [guangdongzhou@126.com](mailto:guangdongzhou@126.com); X. Liang: [liangxq2002@163.com](mailto:liangxq2002@163.com)

**Q. Zhou, M. Hou, B. Bai, Y. Shen, Z. Jia, G. Zhou**

Department of Plastic and Reconstructive Surgery, Shanghai Key Laboratory of Tissue Engineering, Shanghai Ninth People's Hospital, Shanghai Jiao Tong University School of Medicine, Shanghai 200011, China.

**Y. Guo**

Department of Orthopedics, the 80th Group Army Hospital of PLA, Weifang 261000, Shandong province, China.

E-mail: Y. Guo: [gogogyq@126.com](mailto:gogogyq@126.com)

**B. Bai**

Department of Orthopaedics, Qilu Hospital of Shangdong University Centre for Orthopaedics, Advanced Medical Research Institute, Shandong University, Jinan, Shandong 250100, PR China

**Keywords**: osteochondral tissue engineering, vascularization regulation, BMSCs, nanofibrous materials, interface integration


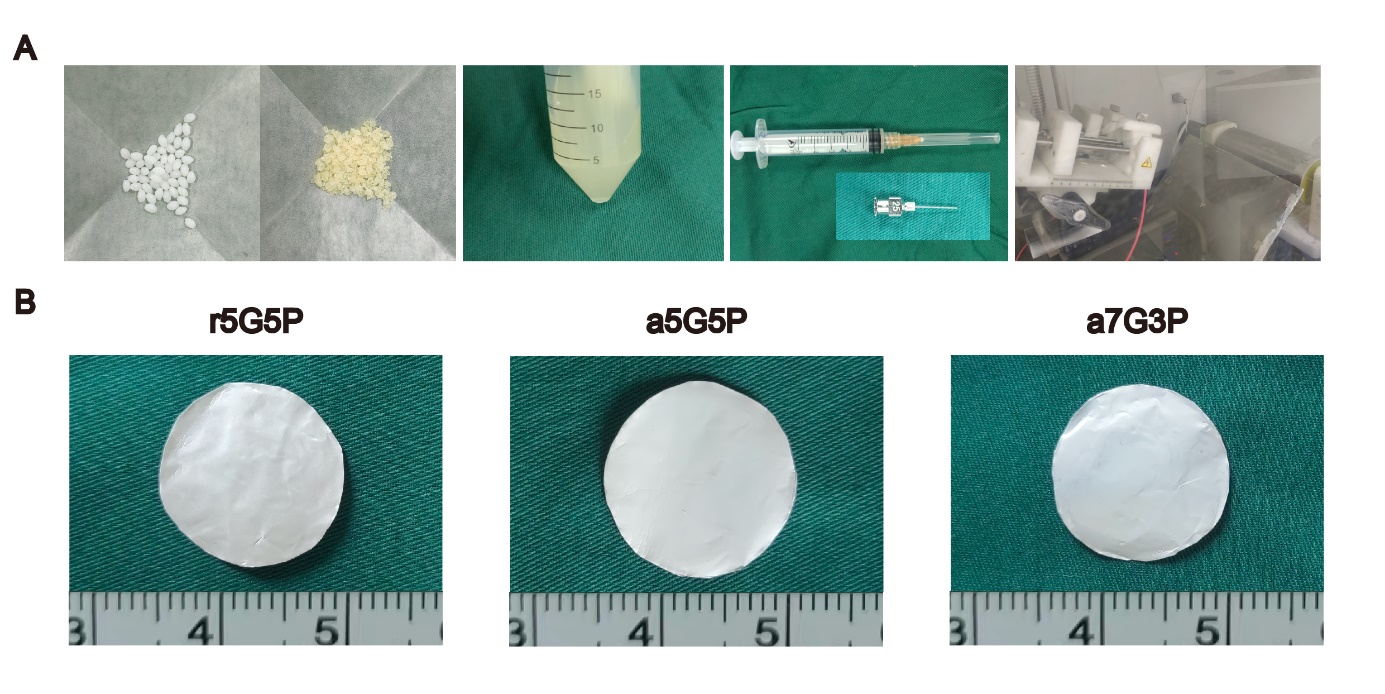


**Figure S1.** Materials and electrospinning process. A) Materials and equipment used for electrospinning nanofibrous membranes. B) Macroscopic view of the three types of nanofibrous membranes.


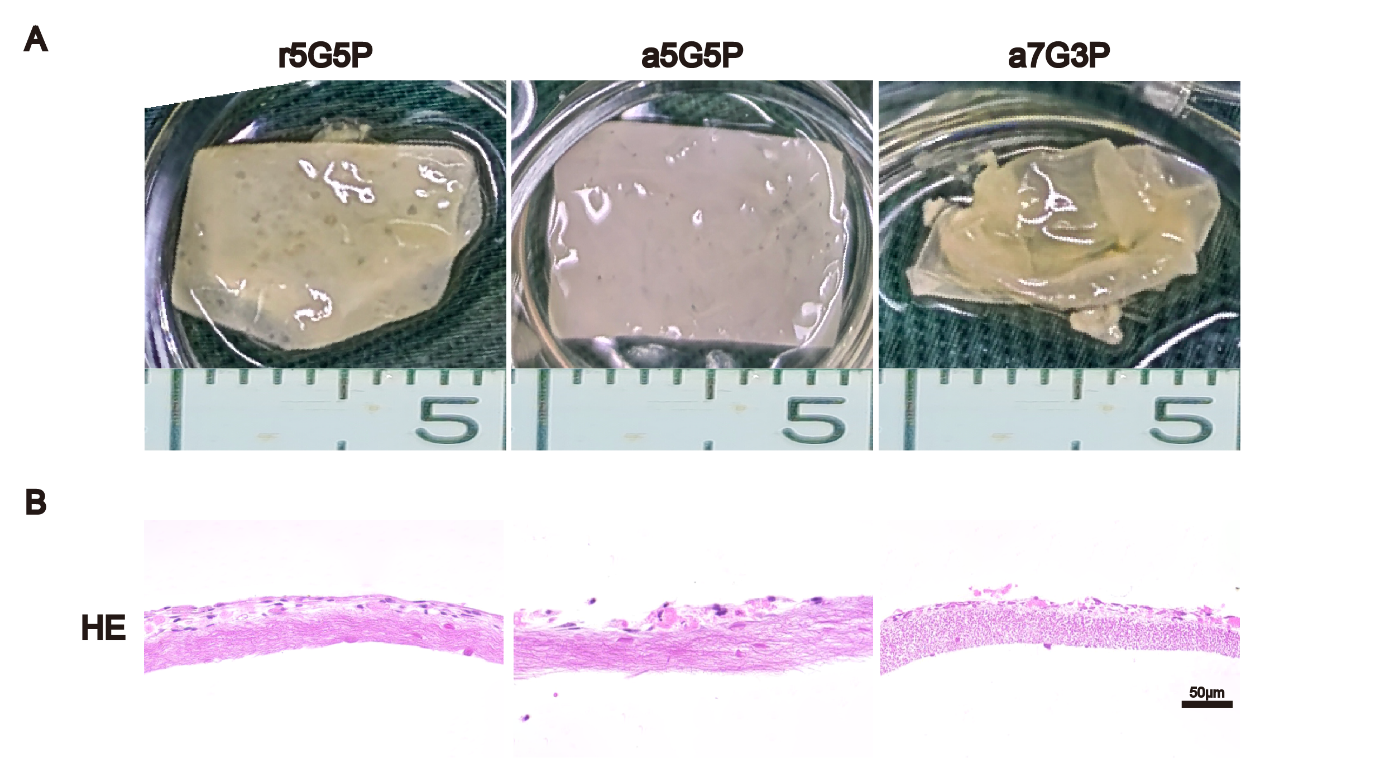


**Figure S2.** Construction process from BMSC-NFM to BMSC-NFMC. A) Process demonstration of rolling and folding BMSC-NFM to form BMSC-NFMC. B) HE staining images of the generated construct after 7 days of in vitro culture.


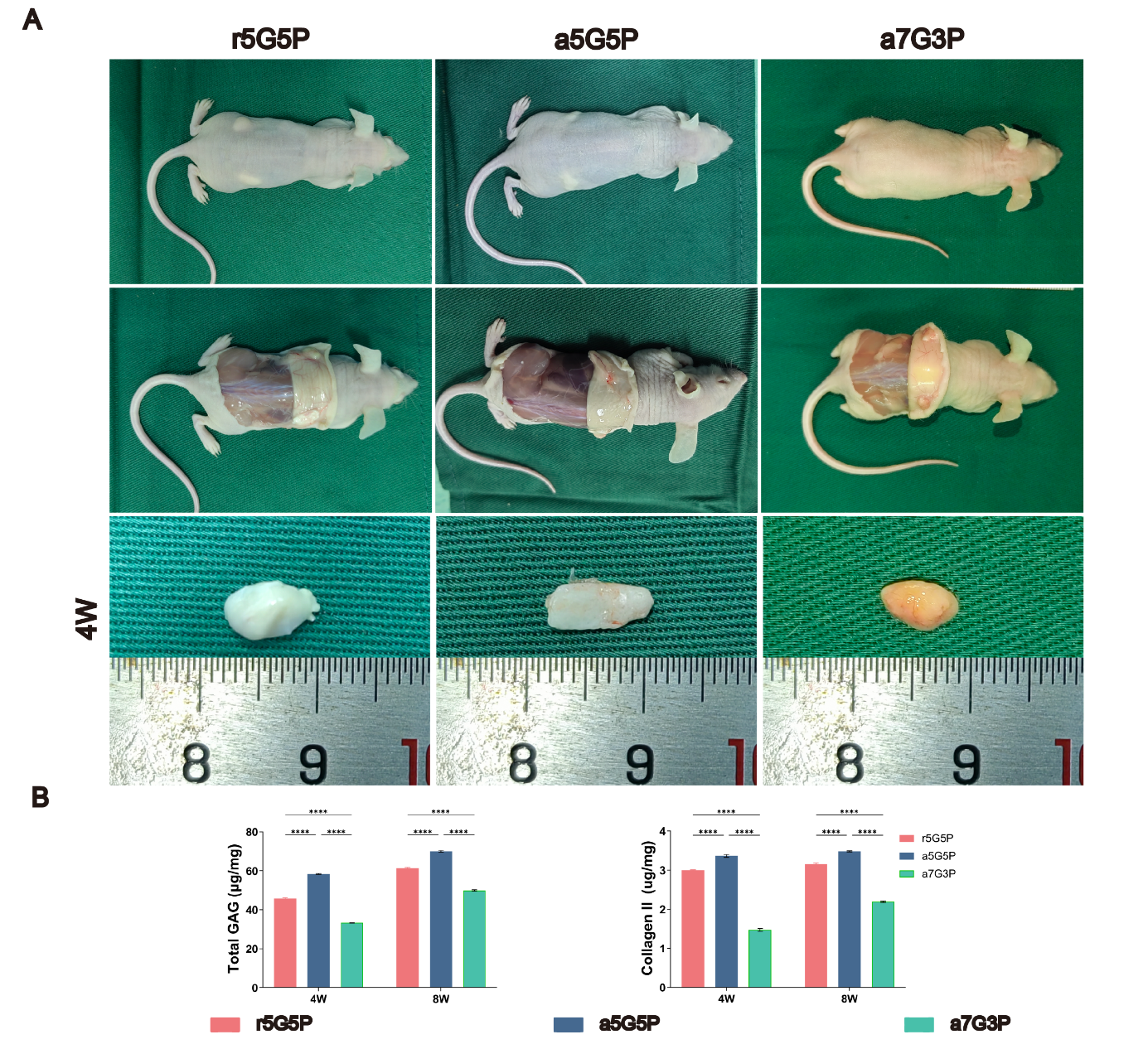


**Figure S3.** A) Macroscopic view of BMSC-NFMC after 4 weeks of subcutaneous implantation in nude mice. B) Quantification of cartilage-specific extracellular matrix components (GAG and collagen type II) in different groups after 4 and 8 weeks of in vivo culture. Data are presented as mean ± SD (n=3 per group). Statistical significance was determined using two-way ANOVA with Bonferroni's post-hoc test. *p<0.05, **p<0.01, ***p<0.001, ****p<0.0001.


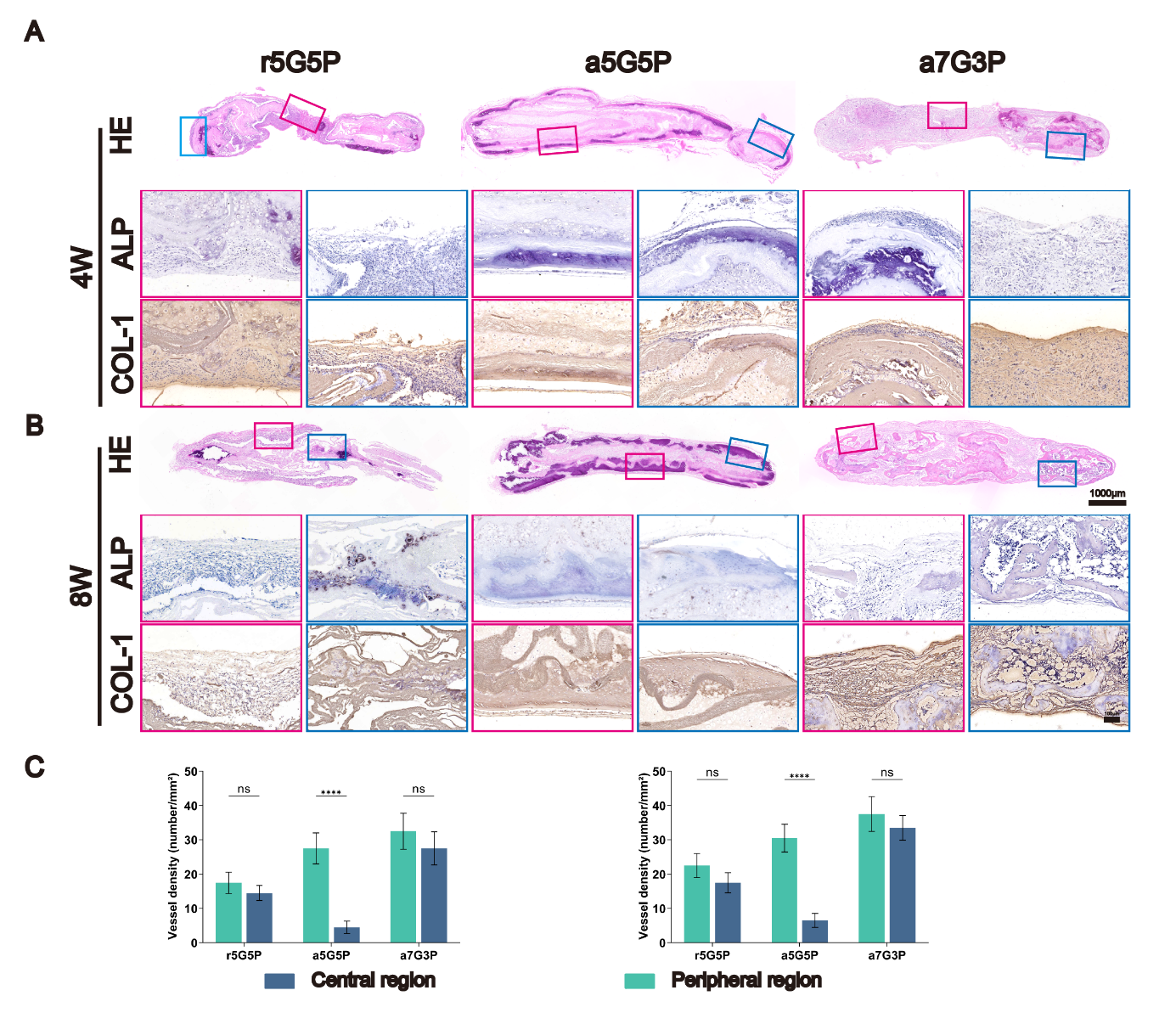


**Figure S4.** A) HE staining, ALP staining, and COL 1 immunohistochemical staining of BMSC-NFMC after 4 weeks of subcutaneous implantation. B) Corresponding staining results after 8 weeks of subcutaneous implantation. C) Quantitative analysis of vessel density in peripheral and central regions at 4 weeks and 8 weeks (n=3, mean±SD, ****: p<0.0001, ns: not significant). Data are presented as mean ± SD (n=3 per group). Statistical significance between central and peripheral regions within each group was determined using paired t-tests. *p<0.05, **p<0.01, ***p<0.001, ****p<0.0001, ns: not ignificant.


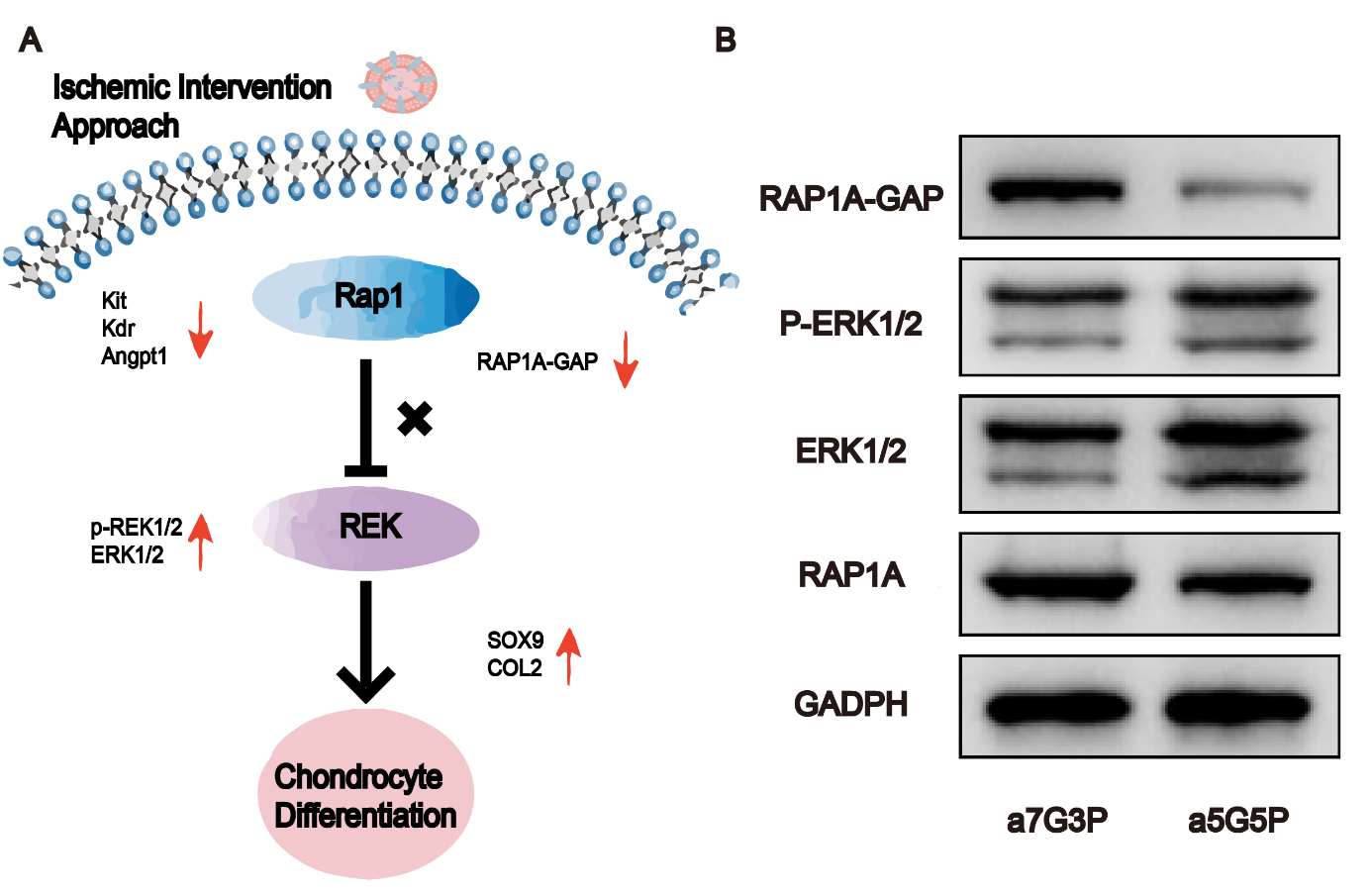


**Figure S5.** A) Schematic diagram showing the molecular mechanism of cartilage formation under ischemic conditions, illustrating the cross-regulation between Rap1 and ERK pathways. B) Western blot analysis of RAP1A-GAP, P-ERK1/2, ERK1/2, RAP1A, and GAPDH in a7G3P and a5G5P groups.


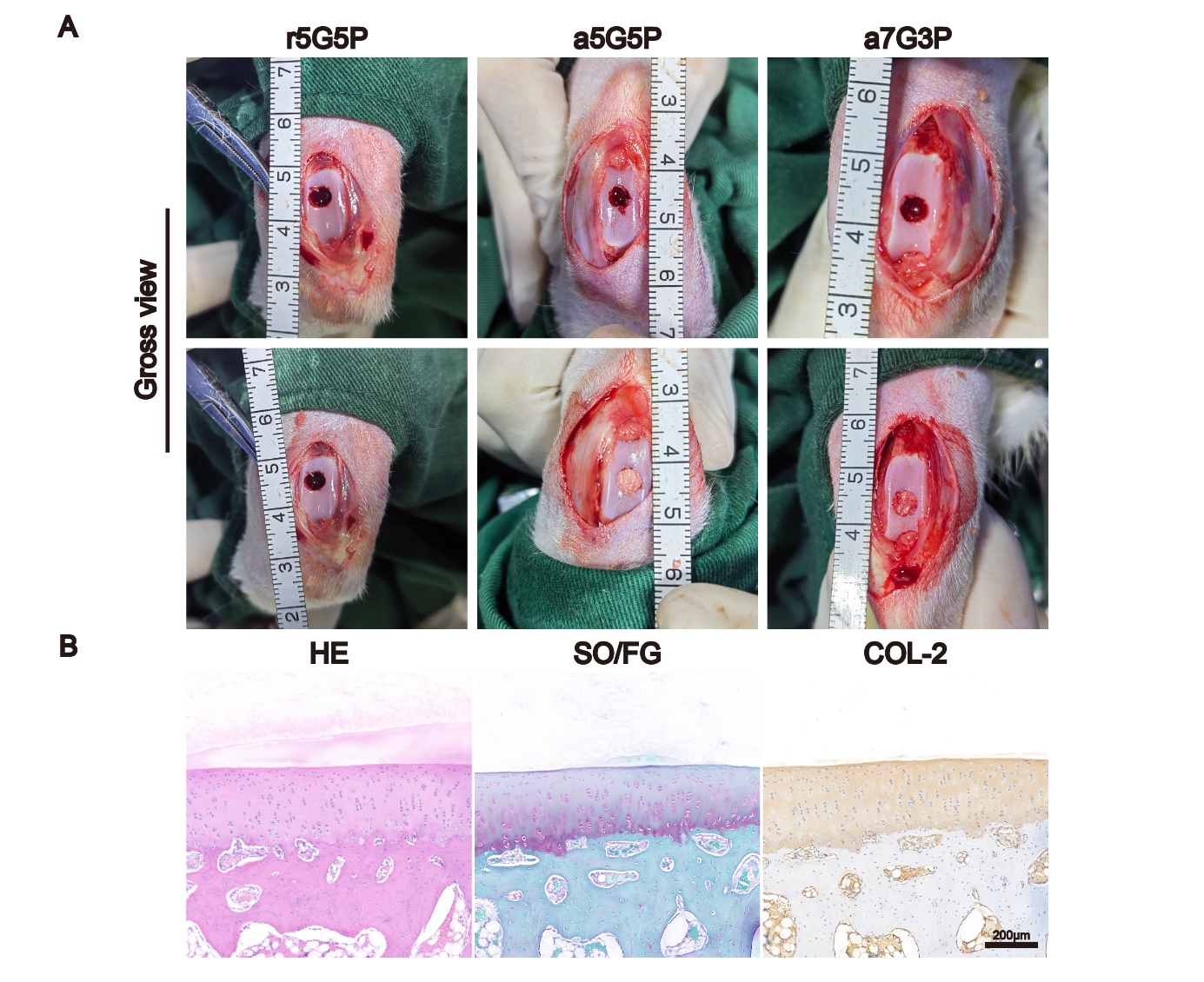


**Figure S6.** A) Surgical procedure for creating and implanting constructs into standardized osteochondral defects in rabbit knee joints. B) Histological evaluation (HE, SO/FG, and COL-2 immunohistochemical staining) of normal rabbit articular osteochondral tissue.

**Table S1.** The primers sequences of qRT-PCR.

| Gene Name | Gene Identifier | Gene Sequence | Product TM  [°C] | Product Length  [bp] |
| --- | --- | --- | --- | --- |
| rabbit ALP  rabbit CD31  rabbit COL2A1  rabbit Runx2  rabbit SOX9  rabbit VEGF  rabbit GAPDH | M0839bf  M0839br  M1398bf  M1398br  M0183f  M0183r  M0837f  M0837r  M0185f  M0185r  M0206cf  M0206cr  M0192f  M0192r | 5' CCT TCA CTG CCA TCC TGT AT 3'  5' GGT AGT TGT TGT GAG CGT AGT C 3'  5' CCC CGA TCC ATT TCA TAG 3'  5' ATC CTG ATG CTG ACT TGA CA 3'  5' TCC TGT GCG ACG ACA TAA TCT 3'  5' GCA GTG GCG AGG TCA GTA G 3'  5' CAG ATG GGA CTG TGG TTA CTG T 3'  5' ACT TGG GGA GGA TTT GTG AA 3'  5' AGG TGC TCA AGG GCT ACG AC 3'  5' TTG ACG TGG GGC TTG TTC T 3'  5' TTA TTT GTA CTG GTT TTT TTG TGT 3'  5' GTT CAG GAT AAG CGA GTG AC 3'  5' ATG GTG AAG GTC GGA GTG A 3'  5' AAC ATC CAC TTT GCC AGA GTT A 3' | 86.3  82.2  87.6  84  87.3  78.6  83.9 | 90  160  103  184  82  87  84 |
